# Molecular screening and genetic diversity of tick-borne pathogens associated with dogs and livestock ticks in Egypt

**DOI:** 10.1371/journal.pntd.0012185

**Published:** 2024-06-05

**Authors:** Haytham Senbill, Donia Karawia, Jehan Zeb, Nouf M. Alyami, Rafa Almeer, Sahidur Rahman, Olivier Sparagano, Aiswarya Baruah

**Affiliations:** 1 Department of Entomology, Faculty of Agriculture, Assam Agricultural University, Jorhat, Assam, India; 2 Department of Applied Entomology & Zoology, Faculty of Agriculture, Alexandria University, Alexandria, Egypt; 3 Department of Pesticide Chemistry & Technology, Faculty of Agriculture, Alexandria University, Alexandria, Egypt; 4 Department of Infectious Diseases and Public Health, Jockey Club College of Veterinary Medicine and Life Sciences, City University of Hong Kong, Kowloon, Hong Kong, China; 5 Department of Zoology, Govt. Ghazi Umara Khan Degree College Samarbagh, Higher Education Department, Khyber Pakhtunkhwa, Pakistan; 6 Department of Zoology, College of Science, King Saud University, Riyadh, Saudi Arabia; 7 Agricultural Sciences and Practice, Royal Agricultural University (RAU), Cirencester, United Kingdom; 8 Department of Agricultural Biotechnology, Faculty of Agriculture, Assam Agricultural University, Jorhat, Assam, India; Connecticut Agricultural Experiment Station, UNITED STATES

## Abstract

**Background:**

The Middle East and North Africa (MENA) offer optimal climatic conditions for tick reproduction and dispersal. Research on tick-borne pathogens in this region is scarce. Despite recent advances in the characterization and taxonomic explanation of various tick-borne illnesses affecting animals in Egypt, no comprehensive examination of TBP (tick-borne pathogen) statuses has been performed. Therefore, the present study aims to detect the prevalence of pathogens harbored by ticks in Egypt.

**Methodology/Principal findings:**

A four-year PCR-based study was conducted to detect a wide range of tick-borne pathogens (TBPs) harbored by three economically important tick species in Egypt. Approximately 86.7% (902/1,040) of the investigated *Hyalomma dromedarii* ticks from camels were found positive with *Candidatus* Anaplasma camelii (18.8%), *Ehrlichia ruminantium* (16.5%), *Rickettsia africae* (12.6%), *Theileria annulata* (11.9%), *Mycoplasma arginini* (9.9%), *Borrelia burgdorferi* (7.7%), *Spiroplasma*-like endosymbiont (4.0%), *Hepatozoon canis* (2.4%), *Coxiella burnetii* (1.6%) and *Leishmania infantum* (1.3%). Double co-infections were recorded in 3.0% (27/902) of *Hy*. *dromedarii* ticks, triple co-infections (simultaneous infection of the tick by three pathogen species) were found in 9.6% (87/902) of *Hy*. *dromedarii* ticks, whereas multiple co-infections (simultaneous infection of the tick by ≥ four pathogen species) comprised 12% (108/902). Out of 1,435 investigated *Rhipicephalus rutilus* ticks collected from dogs and sheep, 816 (56.9%) ticks harbored *Babesia canis vogeli* (17.1%), *Rickettsia conorii* (16.2%), *Ehrlichia canis* (15.4%), *H*. *canis* (13.6%), *Bo*. *burgdorferi* (9.7%), *L*. *infantum* (8.4%), *C*. *burnetii* (7.3%) and *Trypanosoma evansi* (6.6%) in dogs, and 242 (16.9%) ticks harbored *Theileria lestoquardi* (21.6%), *Theileria ovis* (20.0%) and *Eh*. *ruminantium* (0.3%) in sheep. Double, triple, and multiple co-infections represented 11% (90/816), 7.6% (62/816), and 10.3% (84/816), respectively in *Rh*. *rutilus* from dogs, whereas double and triple co-infections represented 30.2% (73/242) and 2.1% (5/242), respectively in *Rh*. *rutilus* from sheep. Approximately 92.5% (1,355/1,465) of *Rhipicephalus annulatus* ticks of cattle carried a burden of *Anaplasma marginale* (21.3%), *Babesia bigemina* (18.2%), *Babesia bovis* (14.0%), *Borrelia theleri* (12.8%), *R*. *africae* (12.4%), *Th*. *annulata* (8.7%), *Bo*. *burgdorferi* (2.7%), and *Eh*. *ruminantium* (2.5%). Double, triple, and multiple co-infections represented 1.8% (25/1,355), 11.5% (156/1,355), and 12.9% (175/1,355), respectively. The detected pathogens’ sequences had 98.76–100% similarity to the available database with genetic divergence ranged between 0.0001 to 0.0009% to closest sequences from other African, Asian, and European countries. Phylogenetic analysis revealed close similarities between the detected pathogens and other isolates mostly from African and Asian countries.

**Conclusions/Significance:**

Continuous PCR-detection of pathogens transmitted by ticks is necessary to overcome the consequences of these infection to the hosts. More restrictions should be applied from the Egyptian authorities on animal importations to limit the emergence and re-emergence of tick-borne pathogens in the country. This is the first in-depth investigation of TBPs in Egypt.

## Author summary

Molecular monitoring of ticks for pathogen nucleic acids is informative when used in the surveillance of diseases transmitted by ticks. In the view of the limited information of genetic aspects of either ticks or their pathogen burdens in Egypt, it is crucial to give more attention into this direction to overcome the consequences might happen due to the caused diseases. We investigated three tick species (*Rhipicephalus rutilus*, *Rh*. *annulatus*, and *Hyalomma dromedarii*). These ticks are well-established in Egypt, infesting dogs, sheep, cattle, and camel among other hosts. The ticks were screened for the presence of different bacterial and protozoan pathogens using PCR technique for four years. The findings showed a prevalence of bacterial agents overall the ticks, followed by the protozoans. Surprisingly, the ticks showed different degrees of co-infections along with single infections. The data provided in this study should be used as a base in the construction of the “One health approach” that is being adopted by the country to protect the lives of both humans and their companion animals.

## Introduction

Egypt probably has the oldest record ever on ticks, as the ancient Egyptians have reported the occurrence of ticks and their infestations, documenting the incidence of tick fever in a figure of a hyaena-like animal’s head found in Dra Abn el-Nago, Western Thebes, dating back to the time of Hatshepsut-Thuthmosis III about 1500 B.C. [1]. Presently, the ticks identified in Egypt include approximately 52 tick species, including eight argasid species infesting birds, rodents, foxes, and hedgehogs [2–7] and 44 ixodid species belongs to the genera of *Amblyomma*, *Haemaphysalis*, *Hyalomma*, *Ixodes*, and *Rhipicephalus*. Apart from the exotic tick species found occasionally in Egypt, the actual fauna includes well-established *Rh*. *sanguineus*, *Rh*. *annulatus*, and *Hy*. *dromedarii* out of 15 *Rhipicephalus* species and 15 *Hyalomma* ticks [8]. Despite the global debate regarding *Rh*. *sanguineus* complex over the past decades, due to the morphological similarity and misidentification between all the species of this complex, molecular techniques have differentiated three distinguished mitochondrial lineages *viz*. temperate lineage, tropical lineage, and southeastern Europe lineage [9–12]. The ambiguous identities of these different lineages have been recently resolved through neotype designations of the temperate lineage to be the actual *Rh*. *sanguineus* [13], followed by a new identification of the tropical lineage to be *Rh*. *linnaei* [14,15], and the southeastern Europe lineage to be dealt with as *Rh*. *rutilus* [16].

Historically, trading routes connecting Africa, Europe, and Asia were being constructed through Egypt due to its strategic location. Egypt’s population (110 million) is notably increasing with anticipated population that is expected to be over 150 million in 2050, rearing and consuming more than 18 million animals in the area, including 5.4 million sheep; 5.1 million cattle, 4 million goats; 3.7 million water buffaloes, 120,000 camels, and 85,000 horses [17]. In view of the universal issues of food security and the rising frequency of undernourished individuals started in 2019, owing mostly to the COVID-19 pandemic [18], the Egyptian authorities were asked to embrace a One Health approach when developing and implementing livestock policies and investments, particularly when dealing with emerging and re-emerging animal diseases, including TBDs that, if left uncontrolled, might jeopardize the entire livestock sector’s development progression. Egypt is expected to grow by 65% in the next three decades. According to the United Nations Population Fund (UNPF), the country’s livestock and animal sector is crucial for its agricultural gross domestic product (GDP) and services, accounting for 40% of the country’s total GDP. However, Egypt faces challenges in providing sufficient meat and livestock products to its 110 million population, affecting its overall agricultural output. Ticks and the diseases they cause are key obstacles that prevent agricultural and animal growth in the country.

Ticks (Acari: Ixodida) are well-known for their diverse and wide capacity for pathogens that could be easily transmitted to their hosts. Although ticks cause direct damage to their hosts during the acquisition of their blood meals (damaging skin and allowing secondary infections to occur), a greater danger comes with their ability to transmit a huge variety of bacteria, viruses, protozoa, and filarial nematodes [19–25]. Tick-borne diseases (TBDs) are a significant constraint on global livestock industries and can result in annual losses amounting to billions of US dollars for farmers [26,27]. As vectors, ticks have grown in prominence among other arthropod groups capable of spreading more pathogens than any other group of invertebrates, causing significant diseases in animals and humans [28,29]. The geographic range of tick species is quickly and continuously expanding globally, probably due to ecological factors and climatic change, demographics, greater human travel, and global animal trafficking/exportations, leading to the revival and redistribution of infectious and zoonotic diseases [30]. In this context, there are two likely scenarios for the emergence and re-emergence of tick-borne diseases (TBDs) in Egypt, which are the migration routes of birds over the African-Eurasian flyways, and the importation of infected animals and their infesting ticks over the barriers of sub-Saharan countries and neighboring ones (Sudan, Libya, Somalia, Ethiopia, Chad, and Kingdom of Saudi Arabia) [31–34], harboring and spreading pathogens that have not been previously identified in the country. Despite the insignificant burden on human populations, cases of zoonotic pathogens in animals that can negatively impact on public health and quality of life, found in ticks and humans have been reported. Generally, human infections due to the TBPs in Egypt are usually underestimated, requiring extensive molecular surveillance to identify known and new pathogens [35]. Limited data from the available Egyptian records of TBP-suffering humans refers to four children in Giza Governorate diagnosed with tick paralysis [36], a couple of human babesiosis cases in Al-Minia Governorate [37,38], four children suffering from Lyme disease in Alexandria Governorate [39], and three human Crimean-Congo hemorrhagic fever (CCHF) cases in Cairo and Gharbia Governorates [40].

Although the Middle East and North Africa (MENA) offer favorable climates and circumstances for tick propagation and dispersion, studies on TBPs in this region are limited [41]. Moreover, despite recent breakthroughs in the characterization and taxonomic justification of numerous tick-borne diseases infecting animals in Egypt, no thorough investigation of TBP statuses has been conducted. In Egypt, scattered information regarding individual groups of TBPs is present, mostly relying on serological detection; however, phenotypic traits have limited value in identifying and delimiting species during microscopic examination [42,43]. Furthermore, TBDs’ symptoms frequently overlap and are widespread, making accurate diagnosis critical for proper treatment and control methods [44]. On the other hand, the emergence of molecular diagnostic tools has led to their increasingly widespread use in studying tick-borne agents due to their high sensitivity and accuracy [45–48]. The continuous advancements in molecular biology techniques have played a great role in the discovery of new species, strains, and genetic variants of microorganisms worldwide, leading to considerable improvements in TBPs’ detection and surveillance [49]. Given the scarcity of non-molecular data on TBDs in Egypt, further molecular research on this topic is required to build a robust dataset on the country’s status and develop appropriate preventative measures.

To effectively manage ticks and their borne-pathogens, comprehensive surveillance studies on circulating tick-borne pathogens in the Egyptian population, molecular diagnostic tools, and enhanced border inspections are necessary. The current study used molecular techniques (PCR-based) to screen for and genetically identify tick-borne pathogens (TBPs) in ticks infesting dromedary camels, cattle, dogs, and sheep from Egypt. This is the first comprehensive study regarding the identification at species level for TBPs circulating in Egypt.

## Methods

### Study location and tick collection

**Fig 1:**
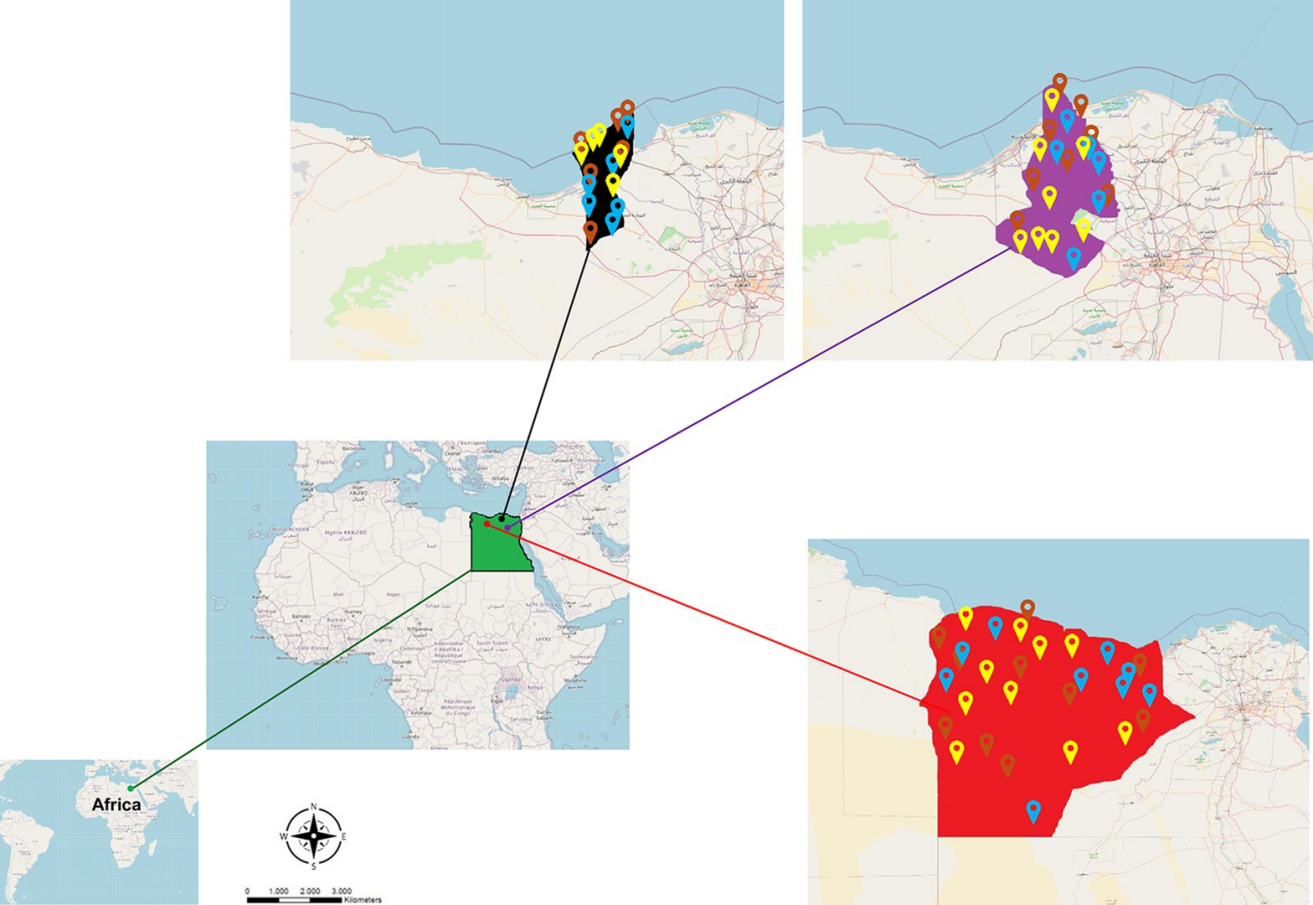
Egypt’s map showing the tick collection spots and their distribution in the study area. Green color refers to Egypt, black to Alexandria governorate, violet to Beheira governorate, and red to Marsa Matrouh governorate. Brown map marks refers to collection sites of *Hyalomma dromedarii*, light blue map marks to *Rhipicephalus annulatus*, and yellow map marks to *Rhipicephalus rutilus*. Open Street Map, which is licensed under an Open Database License ODbL 1.0, was used. The base layer was extracted here: https://www.openstreetmap.org/export. The terms and conditions of the copyright are provided here: https://www.openstreetmap.org/copyright. https://doi.org/10.1371/journal.pntd.0012185.

Egypt is a north African country with over 100 million people and 28 million livestock [50], extends between 22° N and 31.5° N and 25° E and 37° E to cover approximately 1 × 106 km^2^. The annual mean of temperature is between 16°C to 28.6°C with extremes of 5–30°C [51]. The country is divided into 27 governorates and five geographical territories viz. the Great Cairo (Cairo, Giza, and Shoubra El-Kheima), Middle Egypt (Giza, Beni-Suef, Fayoum, and Al-Minia), Middle Delta (Qualiobia, Menoufia, Gharbia, Dakahlia, Kafr Al-Shiekh, and Damietta), East Delta (Sharkia, Port Said, Ismailia, Suez, Siniai), West Delta (Alexandria, Behiera, Nubaria, and Marsa Matrouh), and Upper Egypt (Assuit, Sohag, Qena, Al-Wadi Al-Gadid, and Aswan) [52]. A total of 11,221 Semi and fully engorged adult ticks (females and males; 70%: 30% ratio) were collected between 2017 and 2021 from dromedary camels (2,267), cattle (3,010), dogs (3,355), and sheep (2,589) using tweezers. Tick specimens were collected from three North and North-western Egyptian governorates *viz*. Alexandria (31°12′0.3312′’N; 29° 55′7.4604′’E), Beheira (31°10′48.18′’N; 32°2′5.82′’E), and Marsa Matrouh (31°21′9.7596′’N; 27°15′18.972′’E) (Fig 1). Predominant tick species (over 95% of infestations on specific hosts) have only been considered in the current study (*Hy*. *dromedarii* represented > 96% of camel ticks, whereas ∼ 4% included *Hy*. *excavatum*, *Hy*. *impeltatum* and *Hy*. *franchinii*; *Rh*. *rutilus* represented ∼ 95% of the infested dogs and sheep, whereas only 5% were *Rh*. *turanicus*; *Rh*. *annulatus* were exclusively representing 100% of the ticks infesting cattle). Host-wise collected specimens of every collection trip were kept together based on their morphological characteristics in vials containing 70% ethanol for preservation purposes until further investigation, and data of host type, locality, date, and number of ticks were noted. A list of collected ticks in relation to their hosts is shown in S1 Table. Out of the collected ticks, a total of 3,940 valid specimens of all species were eventually considered and processed in our study to represent the entire tick populations based on their collection locality, collection date and season, host type, tick sex and activity (excluded specimens were due to obstacles of shredded ticks, transfer between countries (Egypt-India), low-yielded DNA specimens, and other biotechnological and sequencing issues).

### Identification of ticks

In our previous study, we identified and characterized the collected tick species at both morphological and molecular levels from brown dog ticks, *Rhipicephalus sanguineus* s.l. (Southeastern-Europe lineage) which was proposed later to be referred as *Rhipicephalus rutilus* (hereafter: *Rh*. *rutilus*), the Texas cattle tick, *Rh*. *annulatus* and the camel tick, *Hyalomma dromedarii*.

### DNA extraction

DNA extraction was carried out using the DNeasy Blood and Tissue Kit (Qiagen, Hilden, Germany) according to the protocol for the purification of total DNA from individual tick species (Approximately 50% of the collected and valid specimens for each tick species). Briefly, the whole tick bodies were cut into pieces using a sterile scalpel blade and homogenized by micro pestles (Jainco Lab, Haryana, India), and DNA was extracted from the homogenate according to the manufacturer’s recommendations. Two hundred μl of Buffer AL and 20 μl of proteinase K were added to the homogenate, and the suspension mixed thoroughly by vortexing using Spinix (Tarsons, Kolkata, India). The total genomic DNA was eluted in a final volume of 30 μl AE buffer, followed by incubation for one minute at room temperature and then centrifugation at room temperature for one minute at 6,000 xg. This last step was repeated with the eluate to increase the DNA yield.

The optical density at 260 and 280 nm was measured with a DNA-RNA calculator (NanoDrop ND-1000, Thermo Fisher Scientific, Waltham, Massachusetts, USA) to quantify DNA content and purity. Genomic DNA was dispensed in aliquots of 10 μl and kept frozen until use in PCR reactions. Prevalence ratios of detected pathogens were based on the results of screened 1,435 *Rh*. *rutilus*, 1,465 *Rh*. *annulatus*, and 1,040 *Hy*. *dromedarii* ticks.

### Polymerase Chain Reaction (PCR)

The oligonucleotide sets of primers used for the amplification of the target genes of every pathogen group, including *Anaplasma* spp., *Babesia* spp., *Borrelia* spp., *Coxiella* spp., *Ehrlichia* spp., *Hepatozoon* spp., *Leishmania* spp., *Mycoplasma* spp., *Rickettsia* spp., *Theileria* spp., *Trypanosoma evansi*, and *Trypanosoma vivax* are shown in Table 1. Every amplification was accompanied by a negative and positive control samples from the same pathogen group (Negative control contained all the PCR reaction components except the template DNA, whereas positive control contained all the PCR reaction components with known template DNA previously recognized from the same pathogen group). For each PCR reaction, 2 μl of 300–500 ng/ μl genomic DNA (gDNA) was used as the template in 50 μl reaction mixture containing 2 μl of each primer concentrated to 10 pmol (forward and reverse); 2 μl of dNTP mixture (2.5 mM); 5 μl of 10X PCR buffer; 0.5 μl of *Takara Ex Taq* polymerase (Takara Bio Inc., Kyoto, Japan); and 36.5 μl of distilled water. The reactions were conducted in GeneAmp PCR System 9700 fast thermal cycler (Thermo Fisher Scientific, Waltham, Massachusetts, USA) with specified conditions for each pathogen group. The obtained PCR products were run by agarose 1% gel electrophoresis later, and every pathogen group sample was loaded into the gel. DL 2,000 DNA marker was used as ladder (Takara Bio Inc., Kyoto, Japan). The bands were finally visualized using the Alpha Innotech AlphaImager EP Gel Imaging System (Bio-Techne, Connecticut, USA).

**Table 1:** Oligonucleotide sets of primers used to detect tick-borne pathogens in this study.

Pathogen	Target gene	Primers	Sequence (5’-3’)	Annealing temperature (°C)	PCR mode	Amplicon size (bp)	Reference
*Anaplasma* spp.	16S rRNA	16ANA-F 16SANA-R	CAGAGTTTGATCCTGGCTCAGAACG GAGTTTGCCGGGACTTCTTCTGTA	69	Touch down: -0.5°C	421	[167]
*Babesia*/*Theileria* spp.	18S rRNA	RIB19 RIB20 BabRumF BabRumR	CGGGATCCAACCTGGTTGATCCTGC CCGAATTCCTTGTTACGACTTCTC ACCTCACCAGGTCCAGACAG GTACAAAGGGCAGGGACGTA	54	Nested	430	[168]
*Theileria ovis*	18S RNA	TSst170F TSst670R TSsr250FN YSsr630RN	TCGAGACCTTCGGGT TCCGGACATTGTAAAACAAA CGCGTCTTCGGATG AAAGACTCGTAAAGGAGCAA	54	Nested	520	[169]
*Babesia bovis*	18S RNA	BoF BoR BoFN BoRN	CACGAGGAAGGAACTACCGATGTTGA CCAAGGAGCTTCAACGTACGAGGTCA TCAACAAGGTACTCTATATGGCTACC CTACCGAGCAGAACCTTCTTCACCAT	55	Nested	356	[170]
*Borrelia* spp.	*flaB*	flaBF flaBR	AACAGCTGAAGAGCTTGGAATG CTTTGATCACTTATCATTCTAATAGC	55	Normal	350	[171]
*Borrelia burgdorferi*	5S-23S ribosomal RNA intergenic spacer region	5S rRNA (rrf) 23S rRNA (rrl) BburgF BburgR	CGACCTTCTTCGCCTTAAAGC TAAGCTGACTAATACTAATTACCC CTGCGAGTTCGCGGGAGA TCCTAGGCATTCACCATA	59	Nested	226–266	[172]
*Coxiella* spp.	16S rRNA	Cox16SF1 Cox16SR2 Cox16SF2 Cox16SR1	CGTAGGAATCTACCTTRTAGWGG GCCTACCCGCTTCTGGTACAATT TGAGAACTAGCTGTTGGRRAGT ACTYYCCAACAGCTAGTTCTCA	56	Nested	719–826	[173]
*Ehrlichia* spp.	16S rRNA	EHR16SD EHR16SR	GGTACCYACAGAAGAAGTCC TAGCACTCATCGTTTACAGC	53	Normal	345	[174]
*Ehrlichia ruminantium*	pCS20	AB130 AB129 AB129 AB128	RCTDGCWGCTTTYTGTTCAGCTAK TGATAACTTGGTGCGGGAAATCCTT TGATAACTTGGTGCGGGAAATCCTT ACTAGTAGAAATTGCACAATCTAT	58	Semi-nested	279	[175]
*Hepatozoon* spp.	18S rRNA	HepF HepR	ATACATGAGCAAAATCTCAAC CTTATTATTCCATGCTGCAG	57	Normal	666	[176]
*Leishmania* spp.	ITS1	LITSR L5.8S	CTGGATCATTTTCCGATG TGATACCACTTATCGCACTT	53	Normal	300–350	[177]
*Mycoplasma*/*Spiroplasma* spp.	16S rRNA	fHF5 rHF6	AGCAGCAGTAGGGAATCTTCCAC TGCACCAACCTGTCACCTCGATAAC	50	Normal	674	[178]
*Rickettsia* spp.	16S RNA	fD1 Rc16S-452n	AGAGTTTGATCCTGGCTCAG AACGTCATTATCTTCCTTGC	54	Normal	416	[179]
*Trypanosoma evansi*	ITS1	Te1F Te1R Te2F Te1R	GCACAGTATGCAACCAAAAA GTGGTCAACAGGGAGAAAAT CATGTATGTGTTTCTATATG GTGGTCAACAGGGAGAAAAT	56	Semi-nested	280	[180]
*Trypanosoma vivax*	Cathepsin L	Tvi2 DTO156	GCCATCGCCAAGTACCTCGCGA TTAGAATTCCCAGGAGTTCTTGATGATCCAGTA	56	Normal	177	[181]

Cases of multiple bands were run in a protocol of gel extraction with the help of QiAquick Gel Extraction Kit (Qiagen, Hilden, Germany). The desired fragments of the expected sizes were extracted from the gel and processed according to the manufacturer’s instructions. The purified DNA was re-analyzed on 1% gel as previously described to check the integrity of the samples.

### DNA Sequence analysis

Amplified amplicons were sent to Bioserve Biotechnologies Pvt. Ltd. (Hyderabad, Telangana state, India) to perform the sequencing based on Sanger sequencing method (dideoxy sequencing or chain termination method) [53]. The protocol used for sequencing the final products started with using 2.5 μl of BigDye Terminator reagent mix (Version 3.1) (Applied Biosystems, California, USA) to be mixed with 1 μl of the template; 0.5 μl of primer; and 1 μl distilled water. In total, 5 μl reaction mixture were used for amplification with specified PCR conditions. The products were run on a Genetic Analyzer ABI3730XL (Applied Biosystems, California, USA). Sequences obtained from the sequence analyzer were analyzed using the GENERUNNER software.

The obtained nucleotide sequences were firstly assembled by the help of CAP3 online tool (http://doua.prabi.fr/software/cap3) (Pôle Rhône-Alpes de Bioinformatique Site Doua (PRABI-Doua), Auvergne-Rhône-Alpes, Lyon, France). The consensus contigs were then aligned and blasted on the National Center for Biotechnology Information (NCBI) using the BLASTn tool (Basic Local Alignment Search Tool) and were run with the parameter of highly similar sequences (Megablast) to inquire the homology between the obtained sequences and the existing sequences on the NCBI database. Thereafter, the contigs were submitted to the GenBank using BankIt submission tool of the National Center for Biotechnology Information (NCBI) (https://www.ncbi.nlm.nih.gov/genbank/)(Bethesda, Maryland, USA) and the accession numbers were obtained. The data were simultaneously made available to the European Nucleotide Archive (ENA) (https://www.ebi.ac.uk/ena) (Hinxton, Cambridgeshire, UK) and to the DNA Data Bank of Japan (DDBJ) (https://www.ddbj.nig.ac.jp/index-e.html) (Shizuoka, Japan).

### Phylogenetic analysis

The sequences were multiply aligned with the sequences of different pathogens obtained from the NCBI database by the MegAlign (DNASTAR, Wisconsin, USA) with default parameter settings. Maximum likelihood (ML) trees were constructed with bootstraps of 1,000 replicates based upon the alignment of the target genes using the Molecular Evolutionary Genetics Analysis Program Ver. X (MEGA X) (Pennsylvania State University, USA). The best-fit evolutionary models were selected according to the lowest values of Bayesian Information Criterion (*BIC*), corrected Akaike Information Criterion (*cAIC*) and maximum likelihood value (*InL*). The evolutionary distances were estimated using the Kimura 2-parameter model [54] for *Ehrlichia* spp. (16S rRNA), *Rickettsia* spp. (16S rRNA) and *Trypanosoma* spp. (ITS1/ Cathepsin L); the Tamura-Nei model [55] for *Anaplasma* spp. (16S rRNA) and *Mycoplasma*/*Spiroplasma* spp. (16S rRNA); the Tamura 3-parameter model [56] for *Borrelia* spp. (FlaB/5S-23S ribosomal RNA intergenic spacer region) and *Leishmania* spp. (ITS1); the Hasegawa-Kishino-Yano model [57] for *Theileria* spp. (18S rRNA), *Eh*. *ruminantium* (pCS20) and *Coxiella* spp. (16S rRNA); and the General Time Reversible (GTR) for *Babesia* spp. (18S rRNA) and *Hepatozoon* spp. (18S rRNA). All ambiguous positions were removed for each sequence pair, and the phylogenetic trees were constructed accordingly.

### Statistical analysis

The obtained datasets were statistically analyzed using MedCalc Statistical Software version 20.006 (MedCalc Software Ltd, Ostend, Belgium; https://www.medcalc.org; 2021). Prevalence rates were calculated as the number of TBP species-infected ticks (individual specimens) divided by the total number of molecularly screened tick specimens of the species. A Kruskal-Wallis test (*post-hoc test*: Conover; *P* < 0.05) was used for the assessment of pathogen prevalence in relation to year of collection, different collection sites, animal hosts, and tick species. The co-infection rates were analyzed by Chi-square to specify any significant associations between the tick species and different degrees of pathogenic co-infections. A confidence interval (CI) of 95% and *P* < 0.05 was considered statistically significant in all analyses.

## Results

### Host-wise distribution of ticks

A total of 11,221 ticks were collected from 498 dromedary camels (n = 2,267), 684 cattle (n = 3,010), 553 dogs (n = 3,355), and 815 sheep (n = 2,589) (S1 Table and S1 Fig). Previous morphological and molecular characterization revealed that the collected ticks belong to two tick genera *viz*. *Rhipicephalus* and *Hyalomma* and three distinct species identified as *Rh*. *rutilus* of dogs and sheep, *Rh*. *annulatus* of cattle, and *Hy*. *dromedarii* of dromedary camels. The other tick species found infesting the animal hosts during this study (less than 4% of *Hy*. *excavatum*, *Hy*. *impeltatum* and *Hy*. *franchinii* infesting camels and *Rh*. *turanicus* for dogs and sheep) were excluded due to their occasional/irregular occurrence and considerably low numbers.

### Detection of pathogens in ticks and prevalence rates

The present study revealed the presence of *Anaplasma marginale*, *Candidatus* Anaplasma camelii, *Babesia canis vogeli*, *Ba*. *bigemina*, *Ba*. *Bovis*, *Theileria annulata*, *Th*. *lestoquardi*, *Th*. *ovis*, *Borrelia burgdorferi*, *Bo*. *theileri*, *Coxiella burnetii*, *Ehrlichia canis*, *Eh*. *ruminantium*, *Rickettsia conorii*, *R*. *africae*, *Hepatozoon canis*, *Leishmania infantum*, *Trypanosoma evansi*, *Mycoplasma arginini*, and *Spiroplasma*-like endosymbionts with an overall infection rate of 84.1% (3,315/3,940) in all the investigated ticks. No single infection was found for *T*. *vivax*. Detailed information about the tick species-pathogen detected in relation to their hosts and GenBank accession numbers are provided in S2 Table along with their similarity, references, and genetic divergence statuses. The occurrence of TBPs in the screened ticks showed significant variations between different tick species (*P* = 0.0433), and between different infested animal hosts (*P* < 0.0346), whereas insignificant differences were found across the collection localities (*P* = 0.0757) and different years of collection (*P* = 0.8031).

### Prevalence of TBPs associated with *Hyalomma dromedarii*

Approximately 86.7% (902/1040) of the screened *Hy*. *dromedarii* ticks were found positive to *Bo*. *burgdorferi*, *Candidatus* Anaplasma camelii, *Eh*. *ruminantium*, *Mycoplasma arginini*, *R*. *africae*, *Spiroplasma*-like endosymbionts, *Th*. *annulata*, *C*. *burnetii*, *H*. *canis*, and *L*. *infantum*. Out of 1,040 screened specimens, *Ca*. A. camelii recorded the highest prevalence by 18.8%, followed by *Eh*. *ruminantium* (16.5%), *R*. *africae* (12.6%), *Th*. *annulata* (11.9%), *M*. *arginini* (9.9%), *Bo*. *burgdorferi* (7.7%), *Spiroplasma*-like endosymbiont (4.0%), *H*. *canis* (2.4%), *C*. *burnetii* (1.6%) and *L*. *infantum* (1.3%) (Table 2 and S2 Fig). Single infections were found in 75 ticks *viz*. *Eh*. *ruminantium* (2.9%), *Th*. *annulata* (2.7%), *R*. *africae* (2.5%), and *M*. *arginini* (0.2%). Double co-infections were recorded in 27 ticks *viz*. *Eh*. *ruminantium* + *R*. *africae* (1.9%) and *Bo*. *burgdorferi* + *M*. *arginini* (1.1%) (Table 3); triple co-infections in 87 ticks by *Ca*. A. camelii + *Eh*. *ruminantium* + *Th*. *annulata* (9.6%). Multiple co-infections by co-existence of four or more pathogens in the same tick were found in 108 individuals *viz*. *Ca*. A. camelii + *Bo*. *burgdorferi* + *M*. *arginini* + *R*. *africae* (5.9%), *Ca*. A. camelii + *L*. *infantum* + *M*. *arginini* + *R*. africae + *Th*. *annulata* (1.4%), *Ca*. A. camelii + *Bo*. *burgdorferi* + *C*. *burnetii* + *Eh*. *ruminantium* + *Spiroplasma* sp. (1.9%), and *Ca*. A. camelii + *Eh*. *ruminantium* + *H*. *canis* + *M*. *arginini* + *Spiroplasma* sp. + *R*. *africae* (2.8%) (Table 4 and S3 Fig).

**Table 2:** Prevalence and species-wise distribution of tick-borne pathogens detected in ticks collected from North and North-Western Egypt.

Samples			Number of positive ticks per pathogen species (%)																			
Tick species	Host	No. of screened ticks (% positive prevalence)	*Anaplasma*		*Babesia*			*Borrelia*		*Coxiella burnetii*	*Ehrlichia*		*Hepatozoon canis*	*Leishmania infantum*	*Mycoplasma/Spiroplasma*		*Rickettsia*		*Theileria*			*Trypanosoma evansi*
			*An*. *mar*	*Ca*. An. cam	*Ba*. *ca*. *vo*	*Ba*. *bi*	*Ba*. *bo*	*Bo*. *burg*	*Bo*. *Th*		*Eh*. *ca*	*Eh*. *ru*			*My*. *arg*	*Sp*.*-like endo*	*R*. *co*	*R*. *af*	*Th*. *lest*	*Th*. *ov*	*Th*. *an*	
*Rhipicephalus rutilus*	**Dogs**	865 (94.3)	–	–	148 (17.1)	–	–	84 (9.7)	–	63 (7.3)	133 (15.4)	–	118 (13.6)	73 (8.4)	–	–	140 (16.2)	–	–	–	–	57 (6.6)
	**Sheep**	570 (42.5)	–	–	–	–	–	–	–	–	–	5 (0.9)	–	–	–	–	–	–	12 3(21.6)	114 (20)	–	–
	**Subtotal**	1,435 (73.7)	–	–	148 (10.3)	–	–	84 (5.9)	–	63 (9.3)	133 (3.1)	5 (0.3)	118 (8.4)	73 (5.1)	–	–	140 (9.8)	–	123 (8.6)	114 (7.9)	–	57 (4.0)
*Rhipicephalus annulatus*	**Cattle** **(Subtotal)**	1,465 (99.2)	312 (21.3)	–	–	266 (18.2)	205 (14.0)	40 (2.7)	187 (12.8)	–	–	37 (2.5)	–	–	–	–	–	181 (12.4)	–	–	127 (8.7)	–
*Hyalomma dromedarii*	**Camels (Subtotal)**	1,040 (86.7)	–	195 (18.8)	–	–	–	80 (7.7)	–	17 (1.6)	–	172(16.5)	25 (2.4)	13 (1.3)	103 (9.9)	42 (4.0)	–	131 (12.6)	–	–	124 (11.9)	–
Total		3,940 (84.1)	312 (7.9)	195 (4.9)	148 (3.8)	266 (6.8)	205 (5.2)	204 (5.2)	187 (4.7)	80 (2.0)	133 (3.4)	214(5.4)	143 (3.6)	86 (2.2)	103 (2.6)	42 (1.1)	140 (3.6)	312 (7.9)	123 (3.1)	114 (2.9)	251 (6.4)	57 (1.4)

***Abbreviations*:** *An*. *mar (Anaplasma marginale)*, *Ca*. *An*. *cam (Candidatus Anaplasma camelii)*, *Ba*. *ca*. *vo*. *(Babesia canis vogeli)*, *Ba*. *bi (Babesia bigemina)*, *Ba*. *bo (Babesia bovis)*, *Bo*. *burg (Borrelia burgdorferi)*, *Bo*. *th (Borrelia theileri)*, *Eh*. *ca (Ehrlichia canis)*, *Eh*. *ru (Ehrlichia ruminantium)*, *My*. *arg (Mycoplasma arginini)*, *Sp-like endo (Spiroplasma-like endosymbiont)*, *R*. *co (Rickettsia conorii)*, *R*. *af (Rickettsia africae)*, *Th*. *lest (Theileria lestoquardi)*, *Th*. *ov (Theileria ovis)*, *Th*. *an (Theileria annulata)*.

**Table 3:** Single and double co-infection rates detected in ticks infesting different hosts in Egypt.

Tick species	Single infections (% of total positive prevalence)													Total	*X^*2*^ = 21.026 *P* = 0.0046	Double co-infections (% of total positive prevalence)								Total	*X^*2*^ = 14.067 *P* = 0.0325
	**Bc**	**Hc**	**Rc**	**Te**	**Tl**	**To**	**Am**	**Bbi**	**Bbo**	**Ra**	**Er**	**Ma**	**Ta**			**Bc+Cb**	**Ec+Rc**	**Hc+Li**	**Tl+To**	**Am+Ra**	**Bbi+Bbo**	**Er+Ra**	**Bb+Ma**		
*Rh*. *rutilus* (Dogs)	**5** **(0.6)**	**49** **(6.0)**	**3** **(0.4)**	**20** **(2.5)**										**77** **(9.4)**		**26** **(3.2)**	**24** **(2.9)**	**40** **(4.9)**						**90** **(11.0)**	
*Rh*. *rutilus* (Sheep)					**45** **(18.6)**	**36** **(14.9)**								**81** **(33.5)**					**73** **(30.2)**					**73** **(30.2)**	
*Rh*. *annulatus*							**39** **(2.9)**	**84** **(6.2)**	**7** **(0.5)**	**7** **(0.5)**				**137** **(10.1)**						**10** **(0.7)**	**15** **(1.1)**			**25** **(1.8)**	
*Hy*. *dromedarii*										**23** **(2.5)**	**26** **(2.9)**	**2** **(0.2)**	**24** **(2.7)**	**75** **(8.3)**								**17** **(1.9)**	**10** **(1.1)**	**27** **(3.0)**	

*Abbreviations*: Am: Anaplasma marginale; Ac: Candidatus Anaplasma camelii; Bc: Babesia canis vogeli; Bbi: Babesia bigemina; Bbo: Babesia bovis; Bb: Borrelia burgdorferi; Bt: Borrelia theileri; Cb: Coxiella burnetii; Ec: Ehrlichia canis; Er: Ehrlichia ruminantium; Hc: Hepatozoon canis; Li: Leishmania infantum; Ma: Mycoplasma arginini; Rc: Rickettsia conorii; Ra: Rickettsia africae; Spi: Spiroplasma endosymbiont; Tl: Theileria lestoquardi; To: Theileria ovis; Ta: Theileria annulata; Te: Trypanosoma evansi.

* Chi-squared *P*-value test was used to assess the association between tick species and the carried pathogens with regard to the co-infection rates, significant association was found at the levels of single infections, and all the levels of co-infections.

**Table 4:** Triple and multiple co-infection rates detected in ticks infesting different hosts in Egypt.

Tick species	Triple co-infections (% of total positive prevalence)							Total	*X^*2*^ = 12.592 *P* = 0.0291	Multiple co-infections (% of total positive prevalence)										Total	*X^2^ = 16.919 *P* = 0.0013
	**Bc+Ec+Li**	**Ec+Hc+Rc**	**Tl+To+Er**	**Am+Ra+Ta**	**Am+Bbi+Bbo**	**Bbi+Bbo+Bt**	**Ac+Er+Ta**			**Bc+Bb+Ec+Rc**	**Bc+Bb+Cb+Rc+Te**	**Am+Bt+Ra+Ta**	**Am+Bbi+Bbo+Bb**	**Am+Bt+Er+Ra**	**Bbo+Bt+Ra+Ta**	**Ac+Bb+Ma+Ra**	**Ac+Bb+Cb+Er+Spi**	**Ac+Li+Ma+Ra+Ta**	**Ac+Er+Hc+Ma+Spi+Ra**		
*Rh*. *rutilus* (Dogs)	**33** **(4.0)**	**29** **(3.6)**						**62** **(7.6)**		**47** **(5.8)**	**37** **(4.5)**									**84** **(10.3)**	
*Rh*. *rutilus* (Sheep)			**5** **(2.1)**					**5** **(2.1)**													
*Rh*. *annulatus*				**29** **(2.1)**	**75** **(5.5)**	**52** **(3.8)**		**156** **(11.5)**				**82** **(6.1)**	**40** **(3.0)**	**37** **(2.7)**	**16** **(1.2)**					**175** **(12.9)**	
*Hy*. *dromedarii*							**87** **(9.6)**	**87** **(9.6)**								**53** **(5.9)**	**17** **(1.9)**	**13** **(1.4)**	**25** **(2.8)**	**108** **(12.0)**	

*Abbreviations*: Am: Anaplasma marginale; Ac: Candidatus Anaplasma camelii; Bc: Babesia canis vogeli; Bbi: Babesia bigemina; Bbo: Babesia bovis; Bb: Borrelia burgdorferi; Bt: Borrelia theileri; Cb: Coxiella burnetii; Ec: Ehrlichia canis; Er: Ehrlichia ruminantium; Hc: Hepatozoon canis; Li: Leishmania infantum; Ma: Mycoplasma arginini; Rc: Rickettsia conorii; Ra: Rickettsia africae; Spi: Spiroplasma endosymbiont; Tl: Theileria lestoquardi; To: Theileria ovis; Ta: Theileria annulata; Te: Trypanosoma evansi.

* Chi-squared *P*-value test was used to assess the association between tick species and the carried pathogens with regard to the co-infection rates, significant association was found at the levels of single infections, and all the levels of co-infections.

### Prevalence of TBPs associated with *Rhipicephalus rutilus*

An overall infection percentage of 73.7% (1,058/1,435) was recorded in *Rh*. *ritulus* ticks. *Babesia canis vogeli*, *Borrelia burgdorferi*, *Coxiella burnetii*, *Ehrlichia canis*, *Hepatozoon canis*, *Leishmania infantum*, *Rickettsia conorii*, *Trypanosoma evansi* in *Rh*. *rutilus* collected from dogs, whereas *Eh*. *ruminantium*, *Theileria lestoquardi*, and *Th*. *ovis* in *Rh*. *rutilus* collected from sheep. Approximately 94.3% (816/865) of *Rh*. *rutilus* tick specimen that were infesting dogs, *Ba*. *canis vogeli* was the predominant species with 17.1%, followed by *R*. *conorii* (16.2%), *E*. *canis* (15.4%), *H*. *canis* (13.6%), *Bo*. *burgdorferi* (9.7%), *L*. *infantum* (8.4%), *C*. *burnetii* (7.3%) and *T*. *evansi* (6.6%) (S4 Fig). For the positive *Rh*. *rutilus* ticks infesting dogs, 77 single infections *viz*. *H*. *canis* (6.0%), *T*. *evansi* (2.5%), *Ba*. *canis vogeli* (0.6%), and *R*. *conorii* (0.4%); 90 double-infected ticks *viz*. *H*. *canis* + *L*. *infantum* (4.9%), *Ba*. *canis vogeli* + *C*. *burnetii* (3.2%), and *Eh*. *canis* + *R*. *conorii* (2.9%) (Table 3); 62 ticks with triple co-infections *viz*. *Ba*. *canis vogeli* + *Eh*. *canis* + *L*. *infantum* (4.0%), and *Eh*. *canis* + *H*. *canis* + *R*. *conorii* (3.6%); and 84 ticks were found co-infected by four or more pathogens *viz*. *Ba*. *canis vogeli* + *Bo*. *burgdorferi* + *Eh*. *canis* + *R*. *conorii* (5.8%) and *Ba*. *canis vogeli* + *Bo*. *burgdorferi* + *C*. *burnetii* + *R*. *conorii* + *T*. *evansi* (4.5%) (Table 4 and S5 Fig).

On the other hand, approximately 42.5% (n = 242/570) investigated *Rh*. *rutilus* specimens collected from sheep were positive for *Th*. *lestoquardi* (21.6%), *Th*. *ovis* (20.0%) and *Eh*. *ruminantium* (0.9%) (S4 Fig). Eighty-one single infections were recorded as *Th*. *lestoquardi* (18.6%) and *Th*. *ovis* (14.9%), whereas 73 double co-infections were found by *Th*. *lestoquardi* + *Th*. *ovis* (30.2%) (Table 3), and five triple co-infections were found by *Th*. *lestoquardi* + *Th*. *ovis* + *Eh*. *ruminantium* (2.1%) (Table 4 and S6 Fig).

### Prevalence of TBPs associated with *Rhipicephalus annulatus*

Approximately 92.5% (1,355/1,465) of *Rh*. *annulatus* ticks were found positive to the infections. *Rhipicephalus annulatus* harbored *Anaplasma marginale*, *Ba*. *bigemina*, *Ba*. *bovis*, *Bo*. *burgdorferi*, *Eh*. *ruminantium*, *R*. *africae*, *Th*. *annulata*, *Bo*. *theileri*. Out of 1,465 screened *Rh*. *annulatus* specimens, *A*. *marginale* was positively found in 21.3% of the ticks, followed by *Ba*. *bigemina* (18.2%), *Ba*. *bovis* (14.0%), *Bo*. *theleri* (12.8%), *R*. *africae* (12.4%), *Th*. *annulata* (8.7%), *Bo*. *burgdorferi* (2.7%) and *Eh*. *ruminantium* (2.5%) (Table 2 and S7 Fig). Single infections were recorded in 137 ticks *viz*. *Ba*. *bigemina* (6.2%), *A*. *marginale* (2.9%), *Ba*. *bovis* (0.5%), and *R*. *africae* (0.5%). Double co-infections were recorded in 25 ticks *viz*. *Ba*. *bigemina* + *Ba*. *bovis* (1.1%) and *A*. *marginale* + *R*. *africae* (0.7%) (Table 3). The cases of triple co-infections were found in 156 ticks *viz*. *A*. *marginale* + *Ba*. *bigemina* + *Ba*. *bovis* (5.5%), *Ba*. *bigemina* + *Ba*. *bovis* + *Bo*. *theileri* (3.8%), and *A*. *marginale* + *R*. *africae* + *Th*. *annulata* (2.1%). The multiple co-infections by four or more pathogens in the same tick were found in 175 ticks *viz*. *A*. *marginale* + *Bo*. *theileri* + *R*. *africae* + *Th*. *annulata* (6.1%), *A*. *marginale* + *Ba*. *bigemina* + *Ba*. *bovis* + *Bo*. *burgdorferi* (3%), *A*. *marginale* + *Bo*. *theileri* + *Eh*. *ruminantium* + *R*. *africae* (2.7%), and *Ba*. *bovis* + *Bo*. *theileri* + *R*. *africae* + *Th*. *annulata* (1.2%) (Table 4 and S8 Fig). Significant associations were found between all the tick species and their harbored pathogens at the levels of single infection, and multiple co-infections (≥ four pathogens). The associations among various tick species and the pattern of infections were found statistically significant (*P* < 0.05) at the levels of single infections (*X*^*2*^ = 21.026, *P* = 0.0046), double co-infections (*X*^*2*^ = 14.067, *P* = 0.0325), triple co-infections (*X*^*2*^ = 12.592, *P* = 0.0291) and multiple co-infections (*X*^*2*^ = 16.919, *P* = 0.0013) (Tables 3 and 4).

### Sequencing, Phylogenetic analysis and genetic diversity

**Fig 2:**
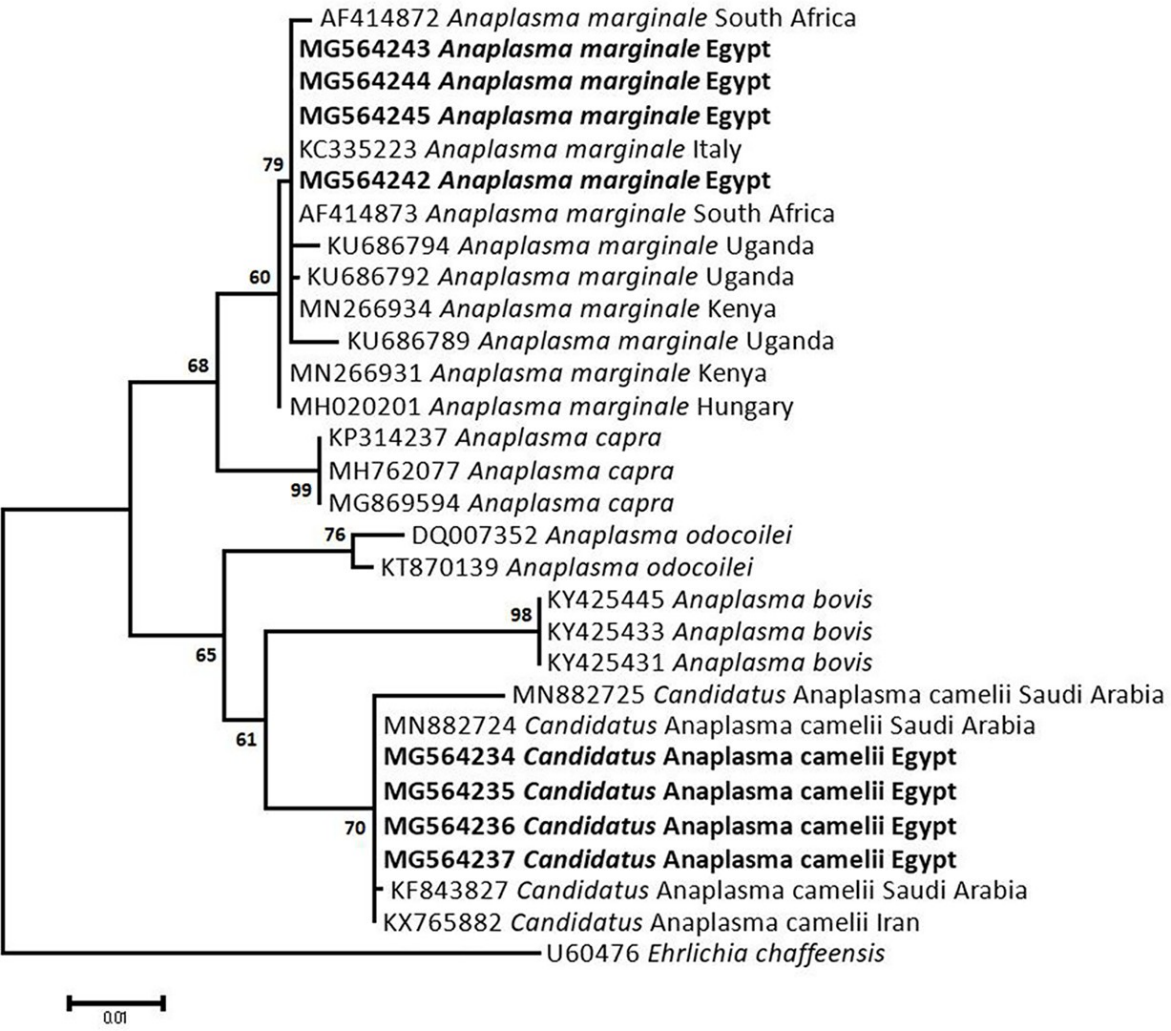
Maximum-likelihood tree based on sequences of the 16S rRNA gene of *Anaplasma* species detected in the present study of Egyptian ticks (bold). Numbers represent bootstrap support generated from 1,000 replications. The tree was constructed with the Tamura-Nei model using *Ehrlichia chaffeensis* as an outgroup.

**Fig 3:**
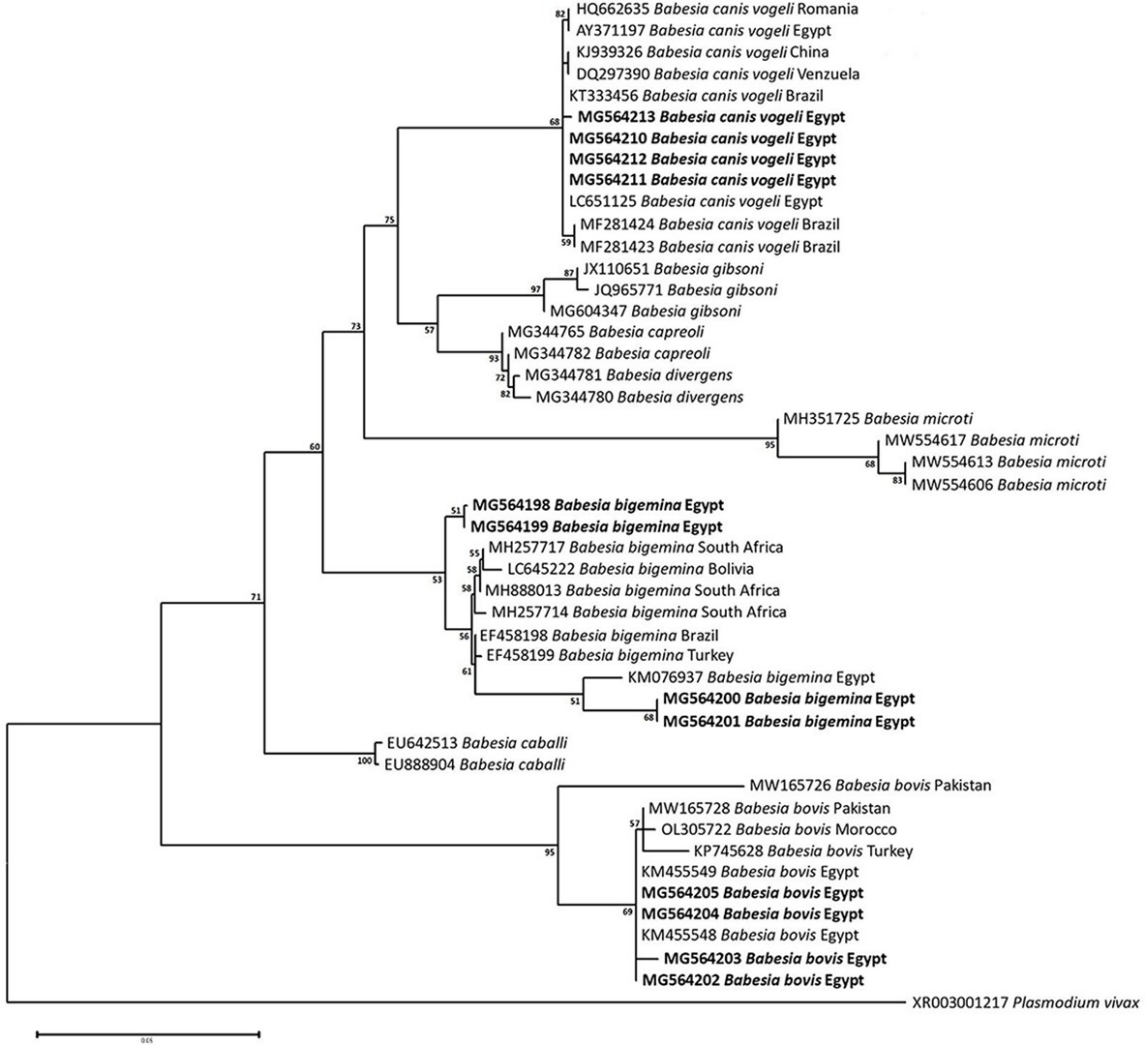
Maximum-likelihood tree based on sequences of the 18S rRNA gene of *Babesia* species detected in the present study of Egyptian ticks (bold). Numbers represent bootstrap support generated from 1,000 replications. The tree was constructed with the General Time Reversible (GTR) model using *Plasmodium vivax* as an outgroup.

**Fig 4:**
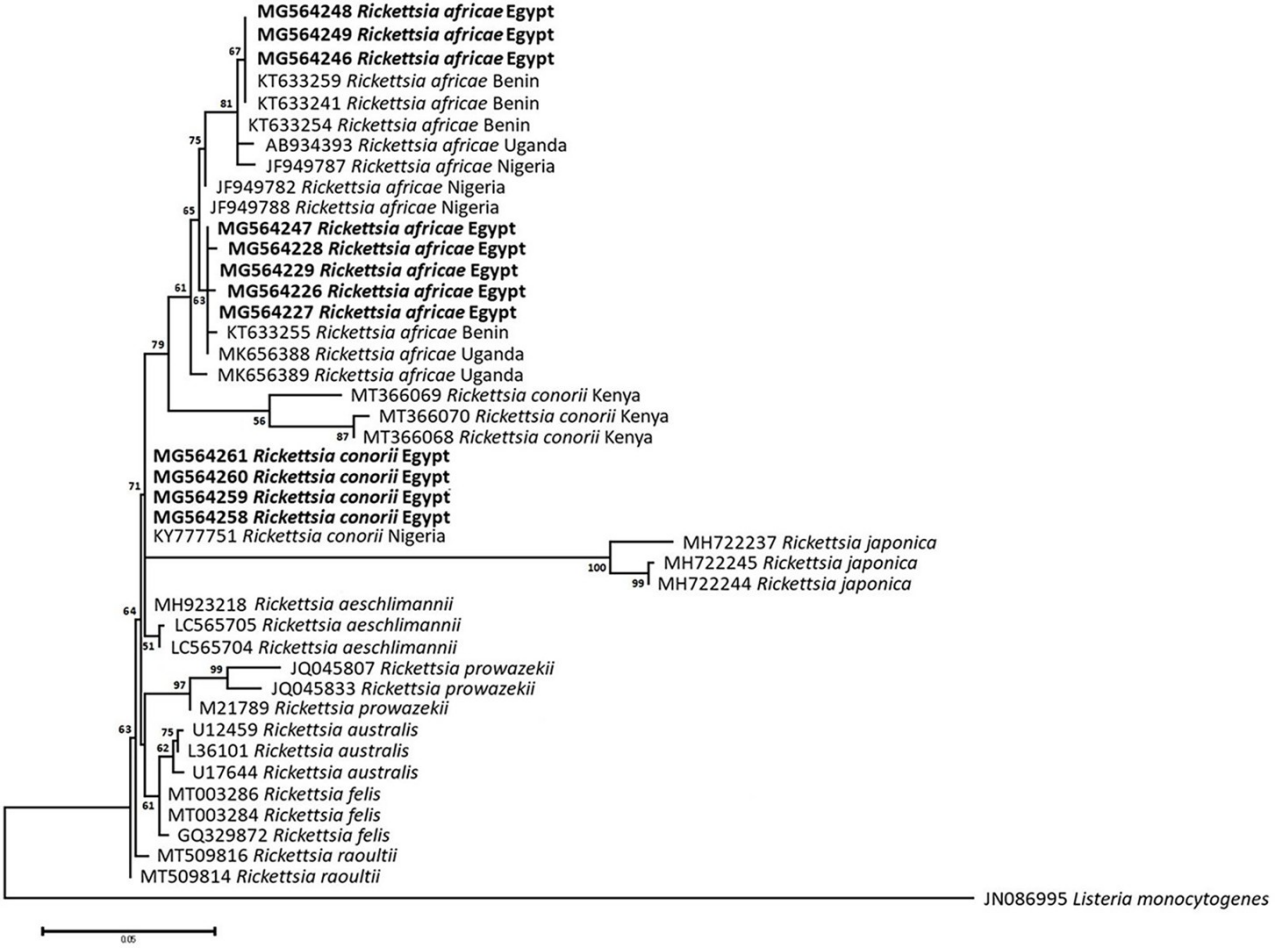
Maximum-likelihood tree based on sequences of the 16S rRNA gene of *Rickettsia* species detected in the present study of Egyptian ticks (bold). Numbers represent bootstrap support generated from 1,000 replications. The tree was constructed with the Kimura 2-parameter model using *Listeria monocytogenes* as an outgroup.

**Fig 5:**
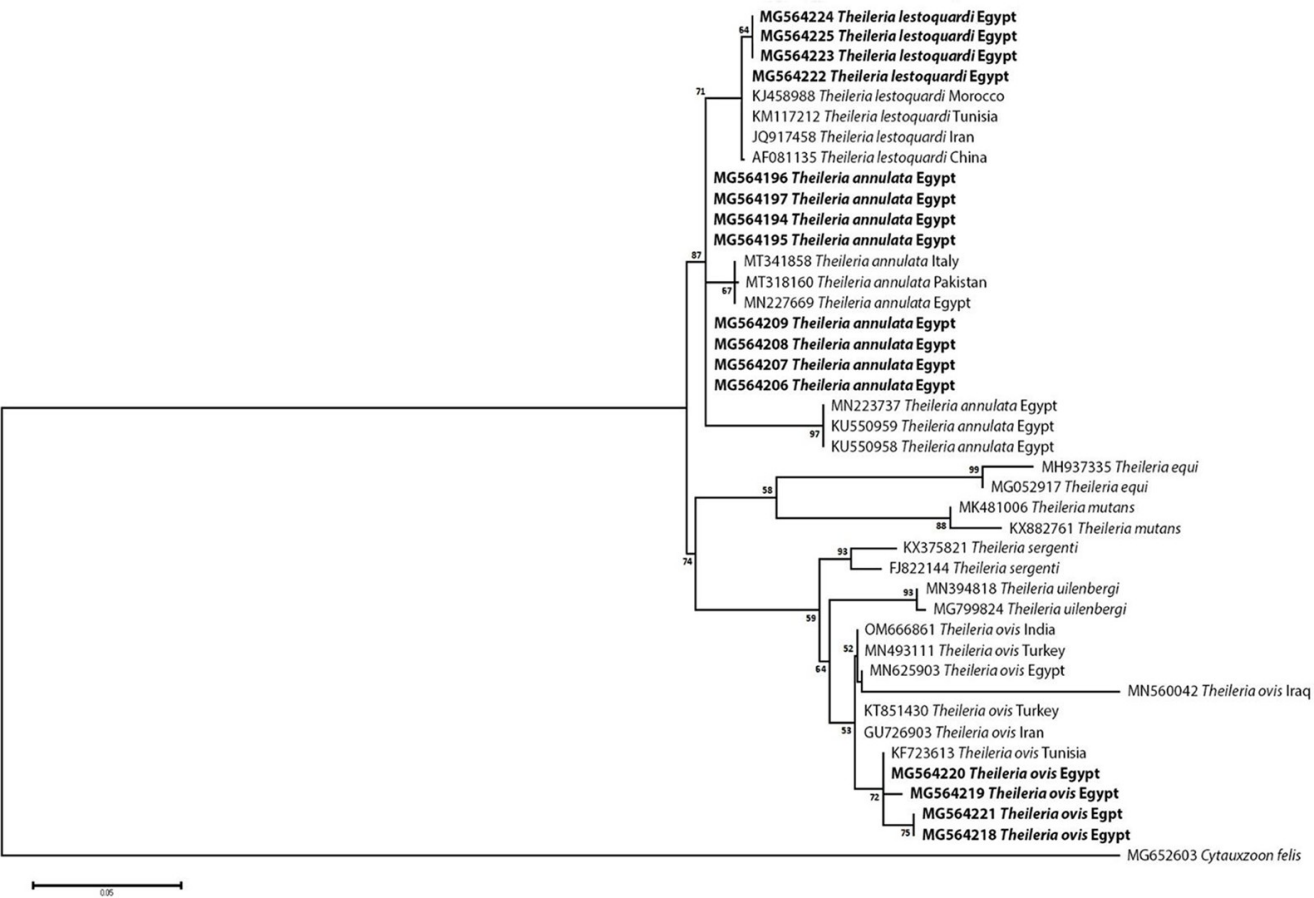
Maximum-likelihood tree based on sequences of the 18S rRNA gene of *Theileria* detected in the present study of Egyptian ticks (bold). Numbers represent bootstrap support generated from 1,000 replications. The tree was constructed with the Hasegawa-Kishino-Yano model using *Cytauxzoon felis* as an outgroup.

**Fig 6:**
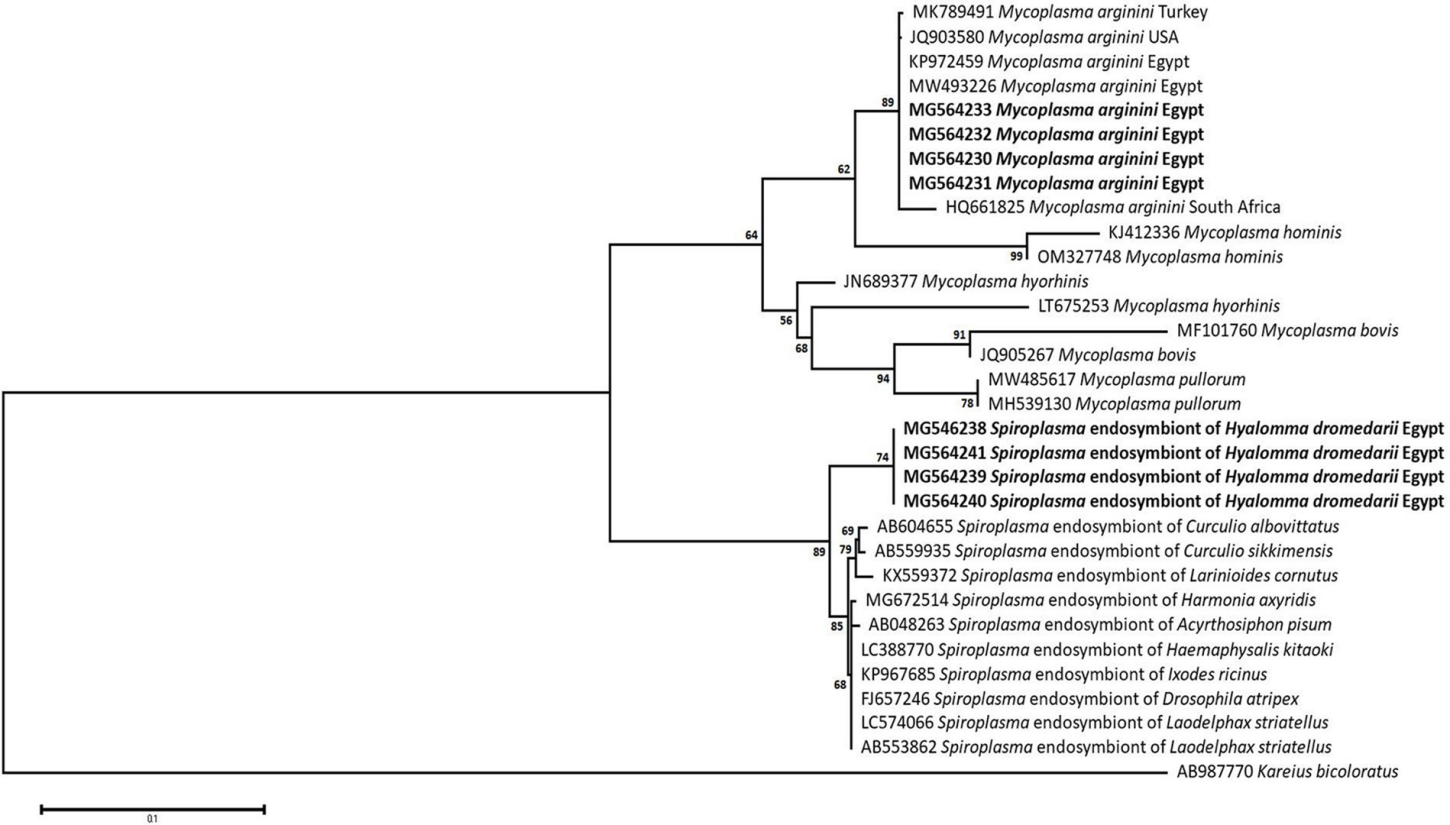
Maximum-likelihood tree based on sequences of the 16S rRNA gene of *Mycoplasma/Spiroplasma* detected in the present study of Egyptian ticks (bold). Numbers represent bootstrap support generated from 1,000 replications. The tree was constructed with the Tamura-Nei model using *Kareius bicoloratus* as an outgroup.

**Fig 7:**
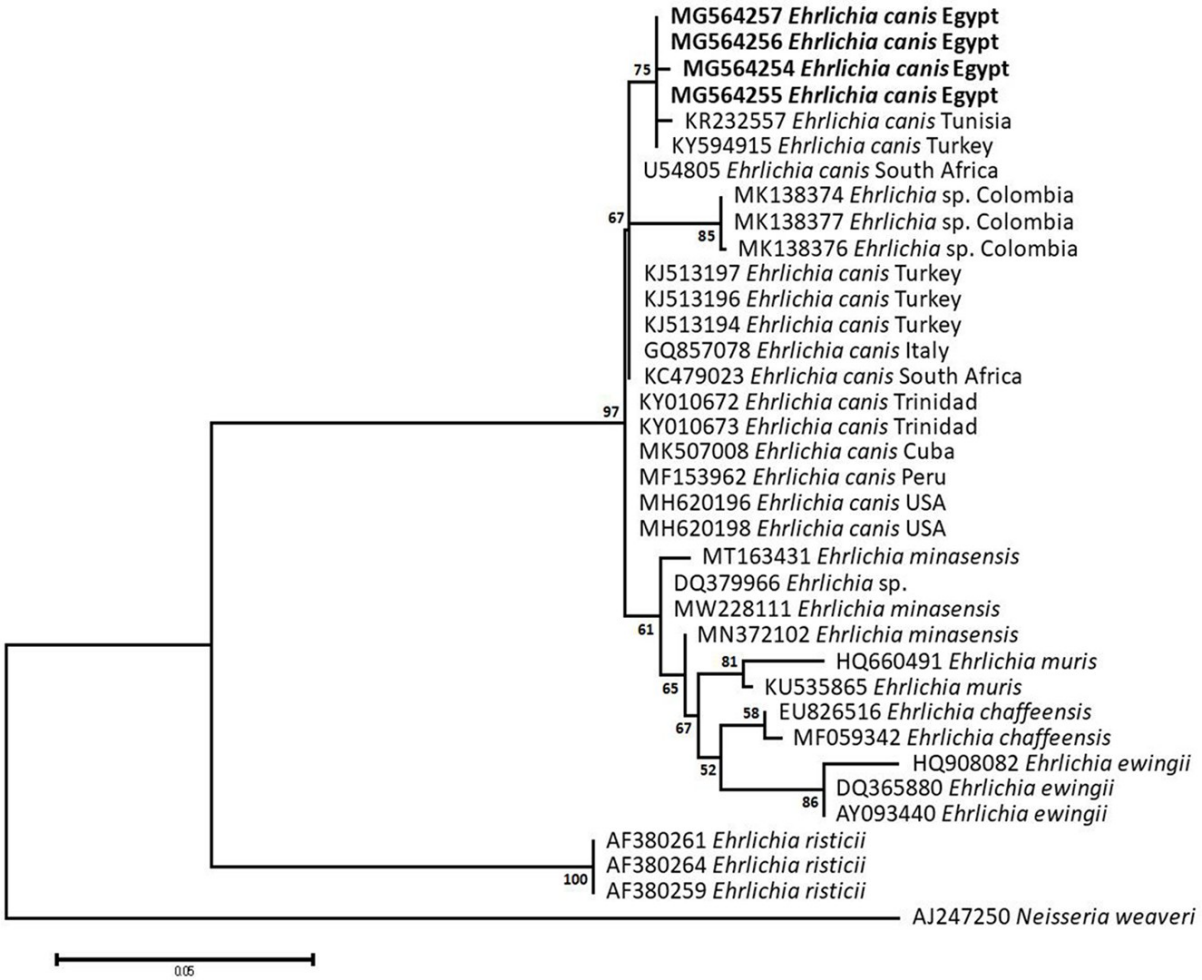
Maximum-likelihood tree based on sequences of the 16S rRNA gene of *Ehrlichia canis* detected in the present study of Egyptian ticks (bold). Numbers represent bootstrap support generated from 1,000 replications. The tree was constructed with the Kimura 2-parameter model using *Neisseria weaveri* as an outgroup.

**Fig 8:**
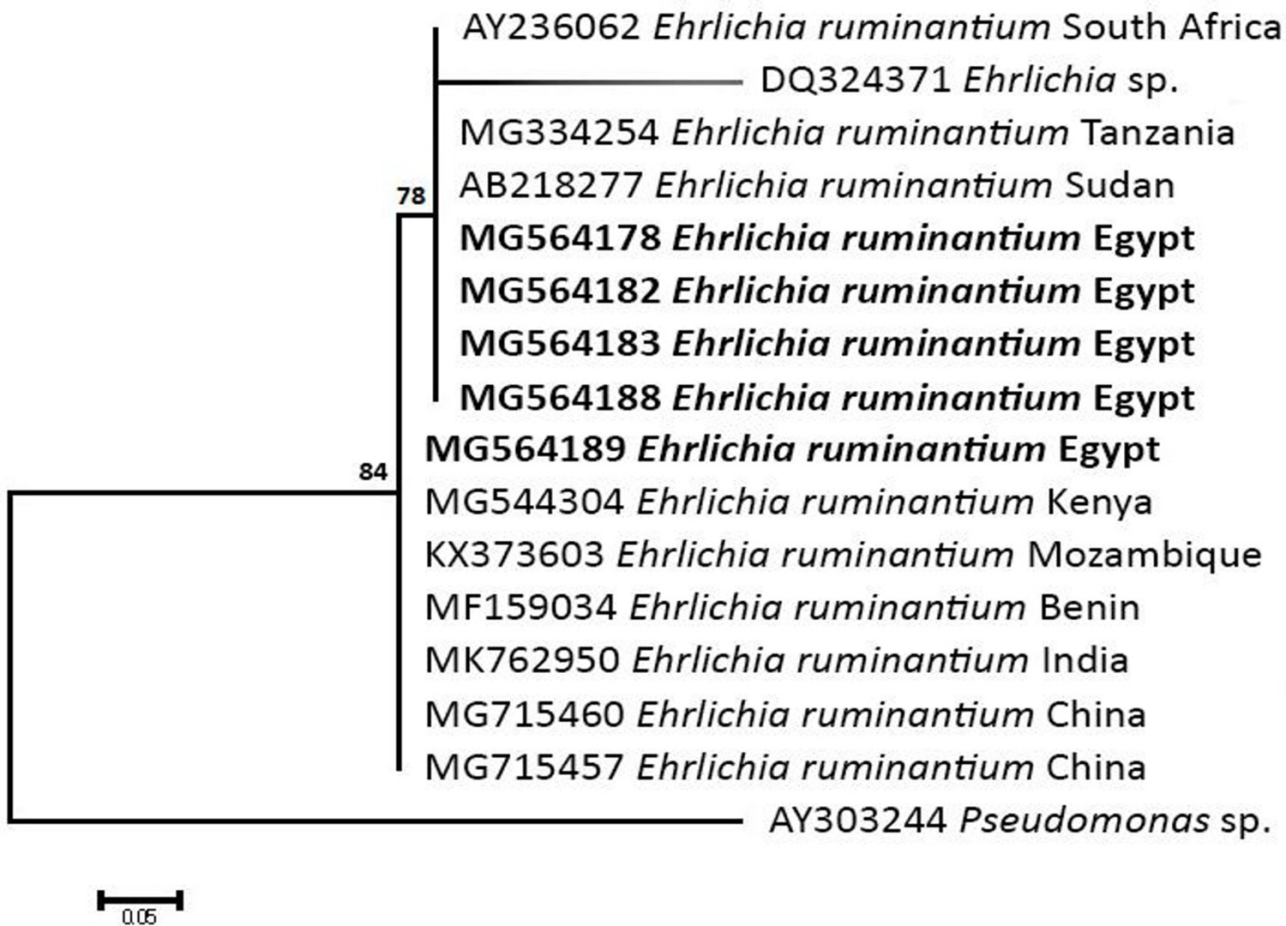
Maximum-likelihood tree based on sequences of the ribonuclease III (pCS20) *gene* of *Ehrlichia ruminantium* detected in the present study of Egyptian ticks (bold). Numbers represent bootstrap support generated from 1,000 replications. The tree was constructed with the Hasegawa-Kishino-Yano model using *Pseudomonas* sp. as an outgroup.

**Fig 9:**
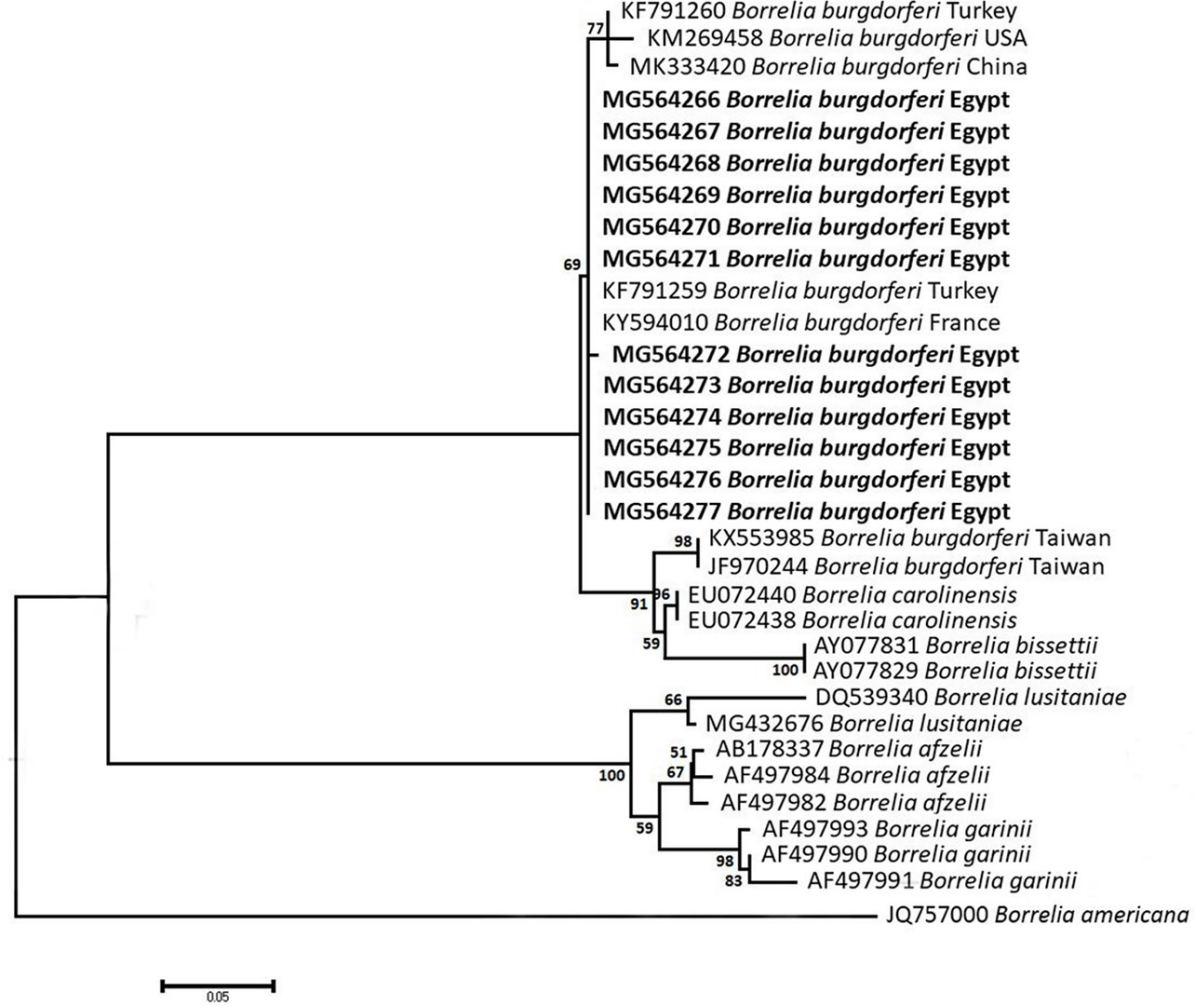
Maximum-likelihood tree based on sequences of the 5S-23S rRNA intergenic spacer of *Borrelia burgdorferi* detected in the present study of Egyptian ticks (bold). Numbers represent bootstrap support generated from 1,000 replications. The tree was constructed with the Tamura 3-parameter model using *Borrelia americana* as an outgroup.

**Fig 10:**
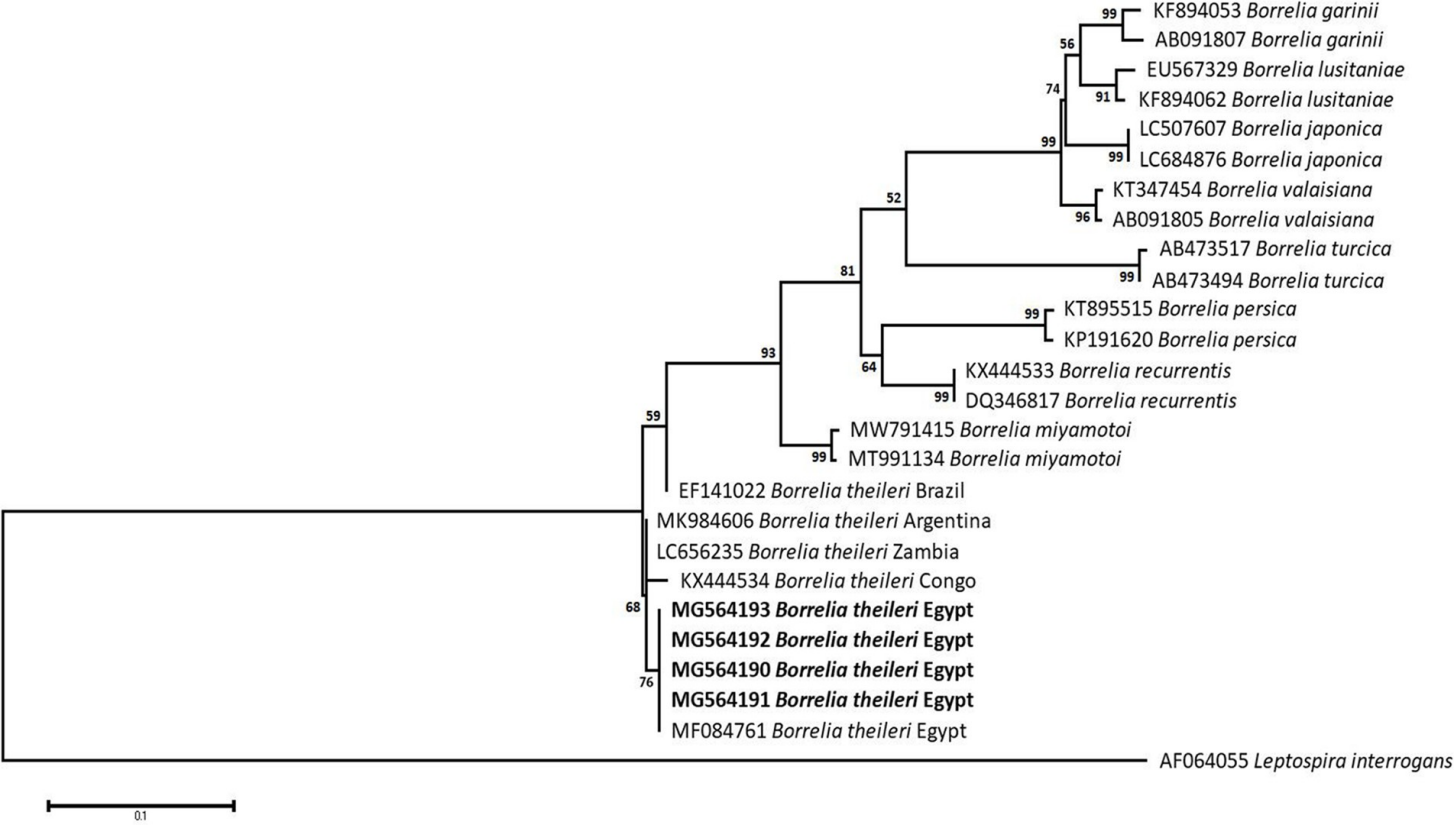
Maximum-likelihood tree based on sequences of the *FlaB* gene of *Borrelia theileri* detected in the present study of Egyptian ticks (bold). Numbers represent bootstrap support generated from 1,000 replications. The tree was constructed with the Tamura 3-parameter model using *Leptospira interrogans* as an outgroup.

**Fig 11:**
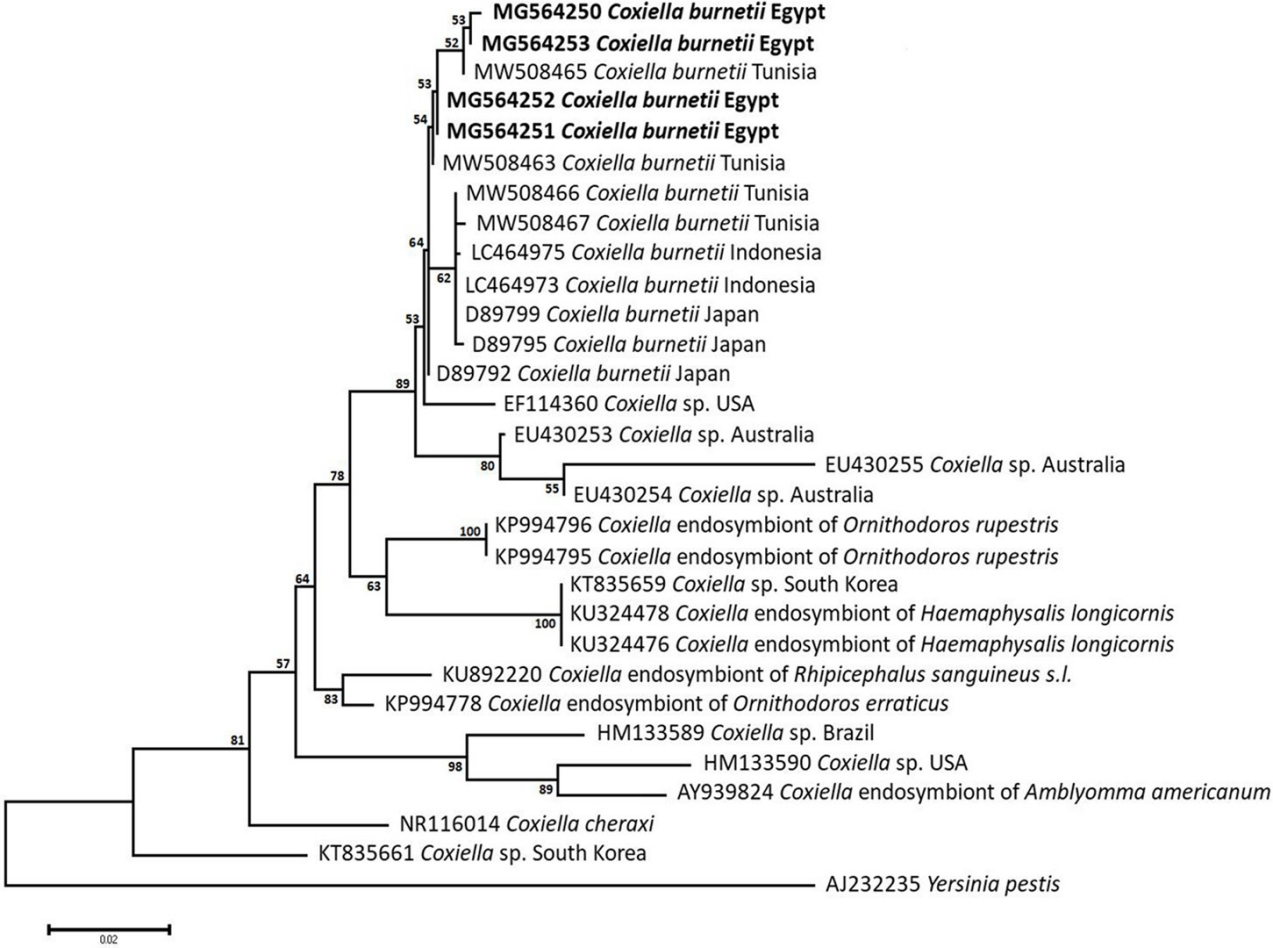
Maximum-likelihood tree based on sequences of the 16S rRNA gene of *Coxiella burnetii* detected in the present study of Egyptian ticks (bold). Numbers represent bootstrap support generated from 1,000 replications. The tree was constructed with the Hasegawa-Kishino-Yano model using *Yersinia pestis* as an outgroup.

**Fig 12:**
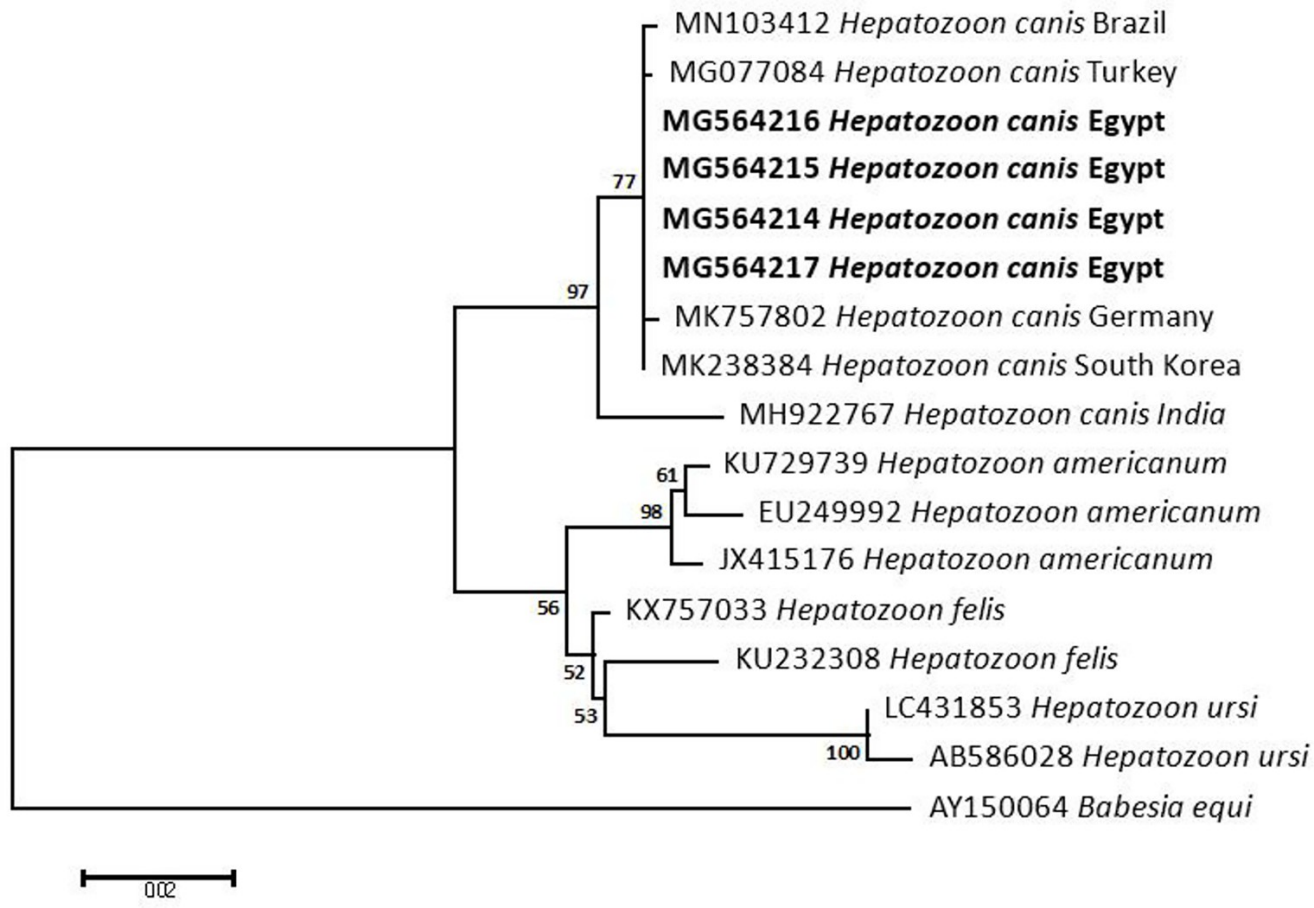
Maximum-likelihood tree based on sequences of the 18S rRNA gene of *Hepatozoon canis* detected in the present study of Egyptian ticks (bold). Numbers represent bootstrap support generated from 1,000 replications. The tree was constructed with the General Time Reversible (GTR) model using *Babesia equi* as an outgroup.

**Fig 13:**
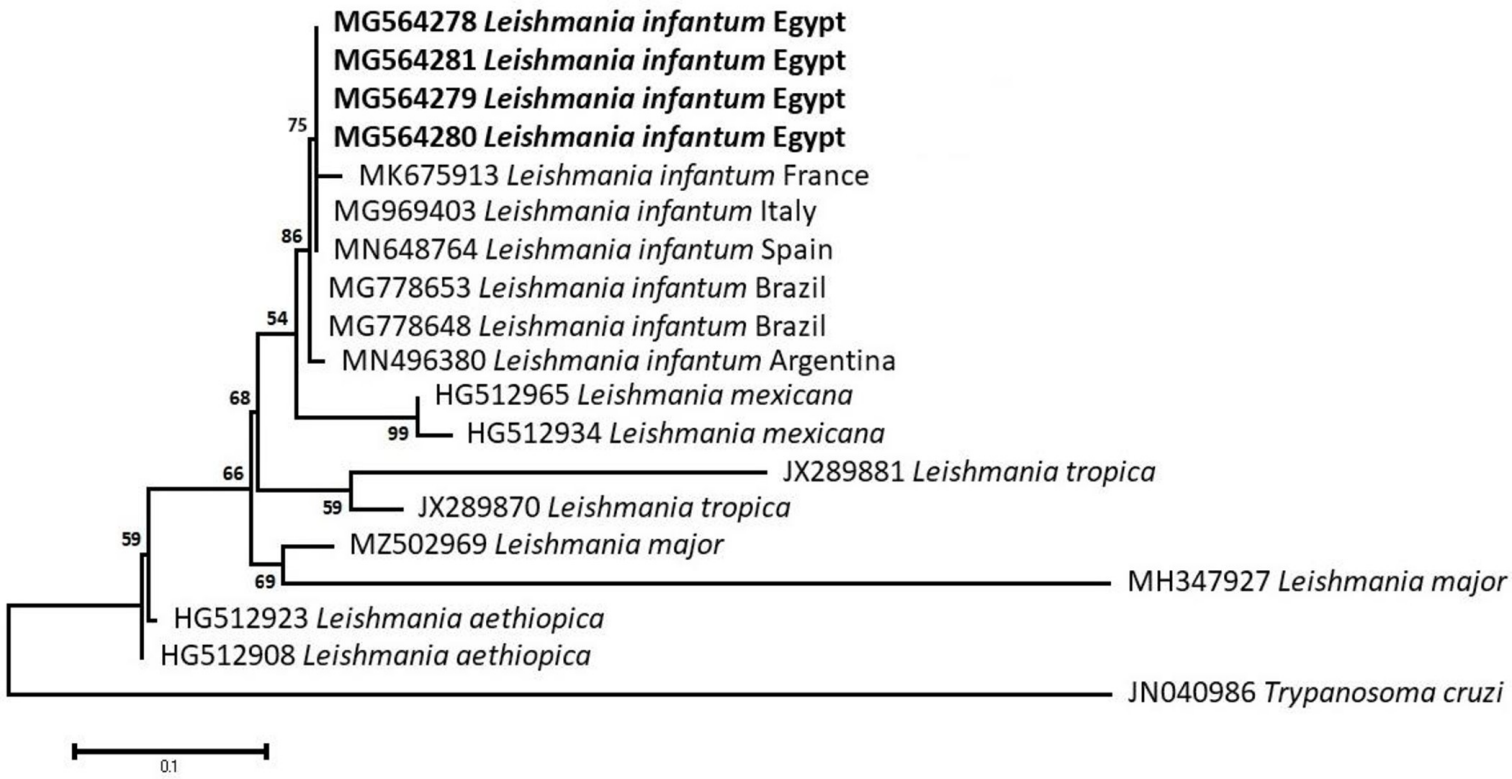
Maximum-likelihood tree based on sequences of the ITS1 gene of *Leishmania infantum* detected in the present study of Egyptian ticks (bold). Numbers represent bootstrap support generated from 1,000 replications. The tree was constructed with the Tamura 3-parameter model using *Trypanosoma cruzi* as an outgroup.

**Fig 14:**
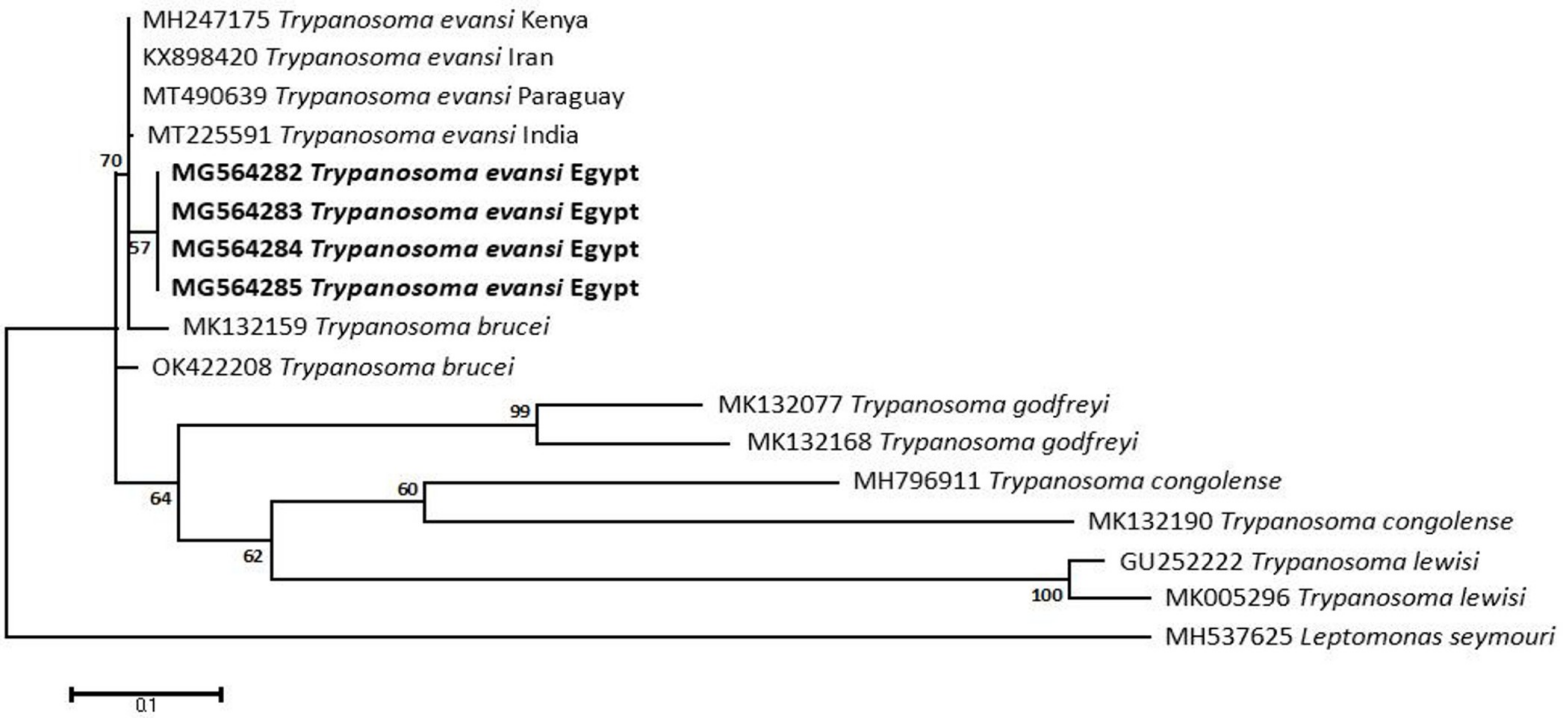
Maximum-likelihood tree based on sequences of the ITS1 gene of *Trypanosoma evansi* detected in the present study of Egyptian ticks (bold). Numbers represent bootstrap support generated from 1,000 replications. The tree was constructed with the Kimura 2-parameter model using *Leptomonas seymouri* as an outgroup.

The obtained sequences of different pathogens detected in this study showed similarities ranging from 98.76 to 100% and a range between 0.001 to 0.009% genetic divergence. Exceptions were given to the *Spiroplasma* endosymbiont that showed similarity between 88.56–100%, and the sequences of *Theileria annulata*, *Leishmania infantum*, and *Mycoplasma arginini* that showed no genetic variations to their references. Details of homology percentages, reference accession numbers, and genetic divergence are shown in S2 Table. Phylogenetic trees of *Anaplasma* spp., *Babesia* spp., *Rickettsia* spp., *Theileria* spp., *Mycoplasma*/*Spiroplasma* spp., *Ehrlichia canis*, *Eh*. *ruminantium*, *Borrelia burgdorferi*, *Bo*. *theileri*, *Coxiella burnetii*, *Hepatozoon canis*, *Leishmania infantum*, and *Trypanosoma evansi* are presented in Figs 2–14. Phylogenetically, Egyptian isolates of *An*. *marginale*, *R*. *africae*, *R*. *conorii*, *Eh*. *ruminantium*, *Bo*. *theileri* clustered with other African isolates from Kenya, Uganda, South Africa, Benin, Nigeria, Tanzania, Sudan, Republic of Congo, Egypt, and Zambia. More closely, isolates of *Ba*. *bovis*, *Ba*. *bigemina*, *Th*. *lestoquardi*, *Th*. *ovis*, and *C*. *burnetii* clustered with isolates from northern Africa such as Tunisia, Egypt, and Morocco. Isolates of *Ba*. *canis vogeli* and some *Ba*. *bigemina* were phylogenetically close to South American isolates from Brazil, Venezuela, Bolivia. Asian and Eurasian isolates from Saudi Arabia, Iran, Pakistan, Turkey, China, India of *Ca*. An. camelii, *Th*. *annulata*, *T*. *evansi* clustered with Egyptian isolates of this study. Egyptian isolates of *M*. *arginini*, *H*. *canis*, and *L*. *infantum* clustered with European and Asian isolates from France, Germany, Spain, and China.

## Discussion

The present study revealed a dominance of tick-borne bacterial agents in Egyptian ticks. The causative agent of bovine anaplasmosis, *A*. *marginale*, prevailed in 21.3% of *Rh*. *annulatus* ticks, whereas the newly emerged *Candidatus* A. camelii was found in 18.8% of *Hy*. *dromedarii* ticks. Reports of the former in Egypt started in 2010, being detected in various hosts in many parts of Egypt [58–70] with the known symptoms of the disease, causing blood impairment that might lead to death [71] (S3 Table). Contrary to the earlier studies, the infected *Rh*. *annulatus* ticks were exclusively infesting cattle hosts in the current study, with a first detection report from Alexandria governorate. This might be due to the high degree of host preference that *Rh*. *annulatus* ticks have. The emergence of *Ca*. A. camelii in Egypt was firstly reported from imported Sudanese camels, followed by only one report from Aswan governorate. Nonetheless, no information is available about its pathogenicity, reservoir hosts, or potential vectors, lowering its zoonotic importance [72]. The present study detected *Ca*. A. camelii in three new localities of Egypt in *Hy*. *dromedarii* ticks, recommending the importance of conducting laboratorial studies regarding the ability of this tick species to transmit *Ca*. A. camelii among different animal hosts and governorates in Egypt, especially that it has been detected once before in Aswan governorate (upper Egypt) [72]. In comparison, previous studies revealed the presence of the human granulocytic anaplasmosis agent, *A*. *phagocytophilum* with a percentage of 7.5% of Egyptian farmers in Giza and Nile Delta. This relatively high infectivity rate may be due to the daily contact of the farmers with the infected animals [73].

In contrast, limited attention has been given to ehrlichiosis in Egypt through ELISA [74,75] or PCR-based few studies [76–78]. Despite the high degree of association between *Eh*. *ruminantium* and *Amblyomma* tick vectors in several African countries, the heartwater disease agent has been found in 16.5% of *Hy*. *dromedarii*, 2.5% of *Rh*. *annulatus*, and 0.3% of *Rh*. *rutilus* ticks of camels, cattle, and sheep, respectively. These findings suggest that animal imports infested by *Amblyomma* ticks, such as *Am*. *variegatum*, *Am*. *gemma*, and *Am*. *lepidum* [79] are likely the main source of the introduction and spread of this pathogen. This is the first report of *Eh*. *ruminantium* in Egypt. Likewise, *Eh*. *canis* was previously detected in several hosts from Central and Northern Egypt [79] (S3 Table); however, the present study confirms its presence in 3.1% of *Rh*. *rutilus* of dogs in Beheira and Marsa Matrouh for the first time, indicating that canine ehrlichiosis cases are expanding throughout the country. In Egypt, transboundary transmission of emerging pathogens or even new genetic variants is mainly due to the importation of animals and birds [80]. *Ehrlichia canis* has not been previously reported in the country until 1995, when its known symptoms appeared among German Shepherd dogs, one of the most popular imported pets in Egypt, along with the Rottweiler and Pit Bull breeds. These imported breeds were later positively detected to be infected by *Ehrlichia* spp. with a prevalence percentage of 8.8%. Since these importations do not receive the proper inspections, they are continuously introducing novel pathogens or new genetic variants of known pathogens into the country. In this context, the increasing pet’s importation into the country might be a reason for the spread and appearance of new genetic variants of *Eh*. *canis*. The human ehrlichiosis seems to be rare in Egypt, even the only investigations regarding the positivity of *Eh*. *chaffeensis* and *Eh*. *ewingii* in humans revealed negative results.

Serological detections of rickettsiosis in Egypt previously revealed positivity in rodents, lambs, and humans to *R*. *conorii* and *R*. *africae* in many parts of the country [81–84]. However, none of these reports has linked the agents of either the spotted fever rickettsioses (SFG) or the neglected African tick-bite fever (ATBF) to any tick species as vectors, despite the familiarity of the former in Mediterranean countries [85]. Thus, the current study links *R*. *conorii* with *Rh*. *rutilus* ticks in three new localities in Egypt, with a prevalence of 9.8%. Furthermore, PCR-based studies have previously revealed the presence of *R*. *africae* in camels, cattle, and their ticks, particularly in North Sinai [85–88], and both *R*. *africae* and *R*. *conorii* in other Egyptian governorates, including Aswan, Sharkia, Cairo, and Giza [89–91] (S3 Table); however, the current study confirms its positivity in 12.6% and 12.4% of *Hy*. *dromedarii* and *Rh*. *annulatus* ticks, respectively, showing the rapid expansion of the disease towards the northern part of the country through the well-establishment of its tick vectors throughout the country.

Despite the typical association between Lyme spirochetes and *Ixodes* tick vectors, causing Lyme spirochetaemia that leads to annual medical costs between USD 712 million and 1.3 billion [92] and led to a total of 23 death cases between 1999 and 2003 in the USA only [93], the present study approved the positivity of 7.7%, 5.9%, and 2.7% of *Hy*. *dromedarii*, *Rh*. *rutilus*, and *Rh*. *annulatus* ticks, respectively. Although *Bo*. *burgdorferi* was previously detected in Egyptian people and their companion animals from different parts, including Fayoum, Beni-Suef, Cairo, and Giza [93,94] (S3 Table), the current study presents molecular evidence of the spirochetes in three new localities of Egypt, suggesting the success of landing migratory birds naturally infested by *Bo*. *burgdorferi*-infected *Ixodes* ticks such as *I*. *ricinus*, *I*. *frontalis*, and *I*. *redikorzevi*, and their co-infestation with local tick species to be the reason behind the emergence of the disease in Egypt. The human cases of Lyme borreliosis in Egypt were only detected once to include two persons and one case in co-infection with *An*. *phagocytophilum*. Since then, no other studies regarding the investigation of Lyme disease in Egyptian people were conducted. Probably, *Bo*. *theileri* is one of the endemic agents of cattle worldwide, particularly in the tropics and subtropics [95–97]. In Egypt, it has been previously detected in bovines from many parts of the country from its main vectors, *Rhipicephalus* ticks (S3 Table). The current study, in context, confirms the presence of bovine borreliosis in the only cattle tick species in Egypt, *Rh*. *annulatus*.

Q fever causative bacteria were previously detected in Egyptian citizens, animals, and ticks such as *Hy*. *dromedarii*, *Hy*. *anatolicum*, *Argas persicus*, *Am*. *variegatum*, *Rh*. *pulchellus*, and *Rh*. *sanguineus*, from Alexandria, Aswan, Cairo, Dakahlia, Giza, Ismailia, Marsa Matrouh, New Valley, Port Said, Sharkia, and Sinai (S3 Table) [98–100]. The disease affects 50 out of 100,000 people annually, with the main influence on the respiratory system [101,102]. The current study revealed the positivity of *Rh*. *rutilus* (9.3%) and *Hy*. *dromedarii* (1.6%) ticks to *C*. *burnetii*. Co-infestation is apparently a major reason behind the expansion of the disease. Seemingly, *M*. *arginini* is a newly emerged agent in Egypt, infecting the genital organs, eyes, and ears of animals and humans [103–105]. The presence of *M*. *arginini* in the blood circulation presents an advantage for ticks harboring it; however, extensive laboratory studies are needed to confirm *Hy*. *dromedarii* as a possible vector or not. Despite the previous reports about the presence of *M*. *arginini* in the blood of infected animal hosts in Giza and Menoufiya governorates [106–108], it has not been linked to tick vectors. Therefore, the present study found 9.9% of *Hy*. *dromedarii* ticks positive to *M*. *arginini* in the first report of linkage to ticks. *Spiroplasma* seems to be a regular presence in various arthropods, which impacts their growth, immunity, reproduction, and speciation [109–111]. Ticks are no exception, as we detected Spiroplasma in *Hy*. *dromedarii* ticks (4%) that shares a high percentage of similarity with previously detected ones, including *S*. *mirum* from *Haemaphysalis leporispalustris* and *S*. *ixodetis* from *Ixodes pacificus*, among others [112–121]. Although we primarily aimed to detect pathogenic microorganisms, we accidentally detected the *Spiroplasma* endosymbionts during the screening of pathogenic *Mycoplasma*, probably due to the similar genetic composition between the two genera. *Spiroplasma* endosymbionts selectively kill the male progeny during embryogenesis, having a female-biased (male-killing) effect on their hosts, which explains the female-biased populations loaded with these endosymbionts throughout our collecting expeditions, whereas no male *Hy*. *dromedarii* was found positive to this microorganism [122].

Protozoal pathogens causing canine and bovine babesiosis were highly abundant in the present study as well, as they were reported in 10.3% and 14–18.2% of *Rh*. *rutilus* and *Rh*. *annulatus* ticks, respectively. To date, eight *Babesia* species, including *Ba*. *canis vogeli*, *Ba*. *bigemina*, and *Ba*. *bovis*, have been reported from Egypt [123–135] (S3 Table). Three new localities have been confirmed for *Ba*. *canis vogeli* in the present study, which was linked to *Rh*. *rutilus* ticks as possible vectors, helping in its expansion since it was detected in dogs in Cairo [135]. Likewise, this study reports *Ba*. *bigemina* and *Ba*. *bovis* from Alexandria for the first time; however, bovine babesiosis seems to be endemic in Egypt.

Bovine theileriosis, initially documented in Egypt in 1947, affects cattle and buffaloes and has been linked to decreased animal production, resulting in annual losses of USD 13.9 to 18.7 billion [136,137]. The current study revealed the presence of *Th*. *annulata* in *Hy*. *dromedarii* (11.9%) and *Rh*. *annulatus* (8.7%) ticks, *Th*. *lestoquardi* (8.6%), and *Th*. *ovis* (7.9%) in *Rh*. *rutilus* of sheep. Although tropical theileriosis was reported from various hosts and governorates [138–145], (S3 Table). Moreover, both *Th*. *lestoquardi* and *Th*. *ovis* were previously reported in Egypt from Aswan, Beheira, Beni-Suef, Cairo, Giza, Menofia, New valley, Qualyobia, Sinai, and Upper Egypt [146–148] (S3 Table); however, no information was available about its possible tick vector. This is the first detection report between *Th*. *ovis*, *Th*. *lestoquardi*, and *Rh*. *rutilus* ticks in Alexandria and Beheira.

In addition, *Hepatozoon canis* is closely linked to its vector, *Rh*. *sanguineus*, worldwide [149]. Although there are characteristic signs, infected hosts usually do not show clinical symptoms [150,151]. Despite earlier serological detections of *H*. *canis* in dog blood or *Rh*. *sanguineus* ticks’ ingested blood meals in Cairo and Giza [152,153] (S3 Table), the present study introduces the first 18S rRNA molecular characterization of *H*. *canis* from Egypt. Likewise, canine leishmaniasis is a zoonotic disease prevalent in the Middle East, North Africa, and the Mediterranean basin, including Egypt, where it was first reported in Egypt over 4,000 years ago, possibly due to trading contacts between ancient Egyptians and Nubia (modern Sudan). The present study confirms the positivity of *Hy*. *dromedarii* (1.3%) and *Rh*. *rutilus* (5.1%) ticks to *L*. *infantum*. Since sand flies of the subfamily Phlebotominae are the only accepted biological vectors for *Leishmania* parasites, the role of ticks as vectors of *Leishmania*, along with the possible transovarial and transstadial transmission routes, should be comprehensively investigated in future studies, at least under laboratory conditions, especially that both the parasite and the ticks have strong associations and interactions in nature [154]. In this context, further experiments are needed to confirm the compatibility of these tick species to transmit the disease. Camel ticks occasionally harbor pathogens related to canines due to the possible co-infestations with dog ticks. African trypanosomiasis, including *T*. *evansi* in camels and other domestic animals, was previously shown to be endemic; however, there have been no reports about its potential transmission to different hosts [155–157]. Thousands of camels are frequently brought into Egypt from its neighbors (Libya, Sudan, and Somalia) for breeding and slaughter. While travelling through Egypt, they interact with indigenous animals at borders, resulting in mixed genotyping and the possibility of strain differences between Egyptian sub-populations and imported strains of *T*. *evansi* [158,159]. Nonetheless, no *Hy*. *dromedarii* ticks were found positive for *T*. *evansi* in our study; however, 4% of *Rh*. *rutilus* were positive, suggesting a possible infection during feeding on other infected hosts. Despite the positivity of the blood samples of the animal hosts to *T*. *evansi* in only three earlier reports from Ismailia and Cairo governorates (S3 Table), more extensive blood investigations from Egyptian animal hosts could be used to complete the route of these infections.

Our findings indicate the presence of multiple TBPs. Considering the correlations between the evolution of host preference, tick biology, and pathogens’ transmission, ticks are apparently following a style of being global generalists and local specialists [160]. In this context, the high degree of host preference of *Rh*. *annulatus* ticks as a one-host tick species for cattle has played a great role in shaping the pathogenic diversity of this tick species exclusively for bovine pathogens. Likewise, *Rh*. *rutilus* ticks infesting sheep were also positive to the ovine pathogens only. On the other hand, the existence of dog hosts guarding the camel farms in the collection sites is probably making a chance for the *Hy*. *dromedarii* ticks to acquire canine pathogens and transmit them into camels, such as *H*. *canis*, *C*. *burnetii*, and *L*. *infantum*, which were positively found in *Hy*. *dromedarii* ticks infesting camels. Co-infections with multiple pathogens are common in Egypt’s livestock due to the mixed farming system, high livestock density, and frequent contact among species, leading to worsening disease severity and a challenge in diagnosing and treating tick-borne diseases clinically [161,162]. The phenomenon has gained attention recently, with diverse pathogenic groups, including protozoa, bacteria, viruses, and variants of the same species, being found [163]. Apparently, ticks can acquire such mixed infections through blood feeding on different vertebrate hosts or through co-feeding, in which the same host acts as a bridge between infected and uninfected ticks, facilitating pathogen transmission [164]. Co-infecting pathogens can interact synergistically through immune modulation, shared siderophores, or genetic exchange, or antagonistically through genotype conflict, resource competition, or cross-immunity, potentially altering disease severity[165]. The scientific community has received warnings about the effects of various pathogenic co-infection statuses; therefore, further research is required to ascertain the true risk that such a combination poses to the host’s health, the agricultural industry, and Egypt’s economy.

Although there is a country-wide risk due to the occurrence of ticks and tick-borne pathogens, the Egyptian agricultural and veterinary authorities are unfortunately dealing with this problem only from an economic point of view when it comes to its large-scale status, neglecting the main reasons behind the beginning of the issue [166]. To date, there is no national surveillance system established in Egypt for TBPs, limiting the available information to only huge incidences regardless the actual occurrence of various pathogens in the country [166]. In view of the outdated records and limited information about the current estimations of animal trade and their tick infestations and TBPs’ infections in Egypt, a series of challenges are present, including real estimations of the country-wide prevalence of TBPs and their burden, particularly in humans, the determination of the genetic divergence, the transmission circulation and dynamics, the evaluation of the economic impact, running successful tick control programs, and performing meta-analysis.

To overcome these obstacles, a package of potential strategies is needed, such as upscaling border inspections of imported animals to prevent the transfer of novel ticks or pathogens into the country, increasing the number of diagnostic laboratories all over the country to guarantee regular screenings of TBPs, and fast policymaking in disease prevention, filling the research gaps in understanding the epidemiological aspects of these pathogens in Egypt. Moreover, the implementation of the One Health approach in research using advanced technologies such as Next Generation Sequencing (NGS) involving ticks, animals, and humans could greatly help in an inclusive view of ticks and their transmitted pathogens in Egypt.

In conclusion, the present study provides an in-depth investigation regarding the pathogenic burdens harbored by three economically important tick species invading Egypt, *viz*. *Rh*. *rutilus* (formerly *Rh*. *sanguineus*, southeastern Europe lineage), *Rh*. *annulatus*, and *Hy*. *dromedarii*, of four types of animals. In addition to the wide range of TBPs detected in the ticks under this study, several novel findings have been recorded for the first time, such as the first records of *A*. *marginale*, *Ba*. *bigemina*, *R*. *conorii*, and *Bo*. *burgdorferi* in new parts of Egypt; new occurrences between *Ca*. A. camelii, *Ba*. *canis vogeli*, *Th*. *ovis*, *Th*. *lestoquardi*, *Eh*. *ruminantium*, and *T*. *evansi* to *Hy*. *dromedarii* and *Rh*. *rutilus* ticks; the first 18S rRNA characterization of *H*. *canis* from Egypt; and the first detection of *M*. *arginini* in ticks and Spiroplasma-endosymbiont in *Hy*. *dromedarii* ticks. Considering the scarce information and studies about ticks and their borne diseases in Egypt, it is difficult to complete the picture of these important and neglected ectoparasites. In this context, laboratory diagnostic capability in the concerned authorities should be increased to facilitate regular screening of tick-borne infections, enhance diagnosis, and inform disease prevention policymaking. Furthermore, research should be rigorously focused on major present gaps in our understanding of the epidemiology of TBDs in Egypt in terms of current trends and directions, such as a coherent One Health approach.

## Supporting information

S1 Table List of tick species and their hosts in the present study in Egypt. (DOCX)

S2 Table Detected pathogens in relation to their tick vectors and hosts. (DOCX)

S3 Table Symptoms and known impact of detected pathogens of this study. (DOCX)

S1 Fig Species-wise amplitude of ticks during the collection trips; (A) *Rhipicephalus rutilus* (Dogs), (B) *Rh*. *rutilus* (Sheep), (C) *Rh*. *annulatus*, (D) *Hyalomma dromedarii*.
(TIF)

S2 Fig Pathogens’ prevalence detected in *Hyalomma dromedarii* ticks of this study (The percentages are out of 1,040 screened ticks). (TIF)

S3 Fig Co-infection overlaps between different detected pathogens in *Hyalomma dromedarii* ticks in Egypt. (TIF)

S4 Fig Pathogens’ prevalence detected in *Rhipicephalus rutilus* ticks infesting dogs (top) and sheep (bottom) of the present study (The percentages are out of 865 and 570 screened ticks of dogs and sheep, respectively).
(TIF)

S5 Fig Co-infection overlaps between different detected pathogens in *Rhipicephalus rutilus* ticks infesting dogs in Egypt. (TIF)

S6 Fig Co-infection overlaps between different detected pathogens in *Rhipicephalus rutilus* ticks infesting sheep in Egypt. (TIF)

S7 Fig Pathogens’ prevalence detected in *Rhipicephalus annulatus* ticks of the present study (The percentages are out of 1,465 screened ticks). (TIF)

S8 Fig Co-infection overlaps between different detected pathogens in *Rhipicephalus annulatus* ticks in Egypt. (TIF)
