# Supplementary material for: Molecular screening and genetic diversity of tick-borne pathogens associated with dogs and livestock ticks in Egypt
Source: PLoS Negl Trop Dis. 2024 Jun 5;18(6):e0012185. doi: 10.1371/journal.pntd.0012185 (PMC11152282; doi:10.1371/journal.pntd.0012185)
Supplement: S1 Table — (DOCX) [file pntd.0012185.s001.docx]

**S1 Table. List of tick species and their hosts in the present study in Egypt**

| Host | | | | | | *Ticks | | | |
| --- | --- | --- | --- | --- | --- | --- | --- | --- | --- |
| Year | **Governorate** | **Species** | | **No of checked animals** | | ***Rhipicephalus rutilus*** | ***Rhipicephalus annulatus*** | | ***Hyalomma dromedarii*** |
| 2017-2018 | **Alexandria** | **Dog** | | 80 | | 412 | – | – | |
|  |  | **Cattle** | | 52 | | – | 311 | – | |
|  |  | **Camel** | | 26 | | – | – | 235 | |
|  |  | **Sheep** | | 44 | | 322 | – | – | |
|  | **Beheira** | **Dog** | | 36 | | 265 | – | – | |
|  |  | **Cattle** | | 64 | | – | 296 | – | |
|  |  | **Camel** | | 13 | | – | – | 62 | |
|  |  | **Sheep** | | 73 | | 283 | – | – | |
|  | **Marsa Matrouh** | **Dog** | | 28 | | 219 | – | – | |
|  |  | **Cattle** | | 30 | | – | 195 | – | |
|  |  | **Camel** | | 66 | | – | – | 291 | |
|  |  | **Sheep** | | 71 | | 85 | – | – | |
| 2018-2019 | **Alexandria** | **Dog** | | 51 | | 345 | – | – | |
|  |  | **Cattle** | | 48 | | – | 361 | – | |
|  |  | **Camel** | | 43 | | – | – | 213 | |
|  |  | **Sheep** | | 25 | | 215 | – | – | |
|  | **Beheira** | **Dog** | | 22 | | 163 | – | – | |
|  |  | **Cattle** | | 68 | | – | 205 | – | |
|  |  | **Camel** | | 12 | | – | – | 125 | |
|  |  | **Sheep** | | 49 | | 136 | – | – | |
|  | **Marsa Matrouh** | **Dog** | | 57 | | 271 | – | – | |
|  |  | **Cattle** | | 39 | | – | 144 | – | |
|  |  | **Camel** | | 61 | | – | – | 224 | |
|  |  | **Sheep** | | 95 | | 288 | – | – | |
| Host | | | ***Ticks** | | | | | | |
| Year | **Governorate** | **Species** | | **No.** | | ***Rhipicephalus rutilus*** | ***Rhipicephalus annulatus*** | | ***Hyalomma dromedarii*** |
| 2019-2020 | **Alexandria** | **Dog** | | 60 | | 344 | – | – | |
|  |  | **Cattle** | | 51 | | – | 212 | – | |
|  |  | **Camel** | | 24 | | – | – | 80 | |
|  |  | **Sheep** | | 52 | | 102 | – | – | |
|  | **Beheira** | **Dog** | | 37 | | 204 | – | – | |
|  |  | **Cattle** | | 95 | | – | 384 | – | |
|  |  | **Camel** | | 46 | | – | – | 164 | |
|  |  | **Sheep** | | 92 | | 361 | – | – | |
|  | **Marsa Matrouh** | **Dog** | | 41 | | 320 | – | – | |
|  |  | **Cattle** | | 43 | | – | 143 | – | |
|  |  | **Camel** | | 61 | | – | – | 365 | |
|  |  | **Sheep** | | 67 | | 194 | – | – | |
| 2020-2021 | **Alexandria** | **Dog** | | 42 | | 314 | – | – | |
|  |  | **Cattle** | | 79 | | – | 366 | – | |
|  |  | **Camel** | | 14 | | – | – | 86 | |
|  |  | **Sheep** | | 88 | | 196 | – | – | |
|  | **Beheira** | **Dog** | | 41 | | 215 | – | – | |
|  |  | **Cattle** | | 77 | | – | 202 | – | |
|  |  | **Camel** | | 43 | | – | – | 147 | |
|  |  | **Sheep** | | 91 | | 352 | – | – | |
|  | **Marsa Matrouh** | **Dog** | | 58 | | 283 | – | – | |
|  |  | **Cattle** | | 38 | | – | 191 | – | |
|  |  | **Camel** | | 89 | | – | – | 275 | |
|  |  | **Sheep** | | 68 | | 55 | – | – | |
| **Total |  | **2,550** | |  |  | **5,944** | **3,010** | **2,267** | |

* Other tick species found during the collection expeditions were excluded from the study due to their irregular occurrence with very low numbers/percentages. For camels: *Hy. excavatum*, *Hy. impeltatum*, and *Hy. franchinii* represented less than 5% of all the collection trips; For dogs and sheep: *Rh. turanicus* represented 4% with occasional occurrences only; For cattle: *Rh. annulatus* was the exclusively sole species found infesting the hosts.

** The total number of checked animal hosts = 2,550 (Dogs = 553, Sheep = 815, Cattle = 684, and camels = 498); the total number of collected *Rh. rutilus* (5,944 ticks; 3,355 from dogs and 2,589 from sheep), *Rh.* *annulatus* (3,010 ticks), and *Hy. dromedarii* (2,267 ticks).
