# Supplementary material for: Molecular screening and genetic diversity of tick-borne pathogens associated with dogs and livestock ticks in Egypt
Source: PLoS Negl Trop Dis. 2024 Jun 5;18(6):e0012185. doi: 10.1371/journal.pntd.0012185 (PMC11152282; doi:10.1371/journal.pntd.0012185)
Supplement: S2 Table — (DOCX) [file pntd.0012185.s002.docx]

**S2 Table. Detected pathogens in relation to their tick vectors and hosts.**

| Host | Tick species | Detected pathogen | Gene | NCBI accession numbers | Reference accession numbers | Reference  Countries | % Similarity | % Genetic divergence |
| --- | --- | --- | --- | --- | --- | --- | --- | --- |
| Dogs | ***Rhipicephalus rutilus*** | *Babesia canis vogeli* | 18S rRNA | MG564210  MG564211  MG564212  MG564213 | HQ662635  AY371197 | Egypt, Romania, China, Venezuela, Brazil | 99.76-100 | 0.002-0.003 |
|  |  | *Borrelia burgdorferi* | 5S-23S rRNA intergenic spacer | MG564274  MG564275  MG564276  MG564277 | MK333420  KY594010 | Turkey, China, USA, France | 99.59-100 | 0.003-0.004 |
|  |  | *Coxiella burnetii* | 16S rRNA | MG564251  MG564252 | MW508465  MW508463 | Tunisia, Indonesia, Japan | 99.72-100 | 0.001-0.002 |
|  |  | *Ehrlichia canis* | 16S rRNA | MG564254  MG564255  MG564256  MG564257 | KR232557  KY594915 | Tunisia, Turkey, South Africa | 99.75-100 | 0.002-0.003 |
|  |  | *Hepatozoon canis* | 18S rRNA | MG564214  MG564217 | MG077084  MK757802 | Brazil, Turkey, Germany, South Korea | 99.83-100 | 0.001-0.002 |
|  |  | *Leishmania infantum* | ITS1 | MG564279  MG564280 | MK675913  MG969403 | France, Italy, Spain | 100 | Nil |
|  |  | *Rickettsia conorii* | 16S rRNA | MG564258  MG564259  MG564260  MG564261 | KY777751  MT366068 | Nigeria, Kenya | 99.79-100 | 0.003-0.005 |
|  |  | *Trypanosoma evansi* | ITS1 | MG564282  MG564283  MG564284  MG564285 | MT225591  MT490639 | Kenya, India, Paraguay, Iran | 99.66-100 | 0.003-0.005 |
| Sheep | ***Rhipicephalus rutilus*** | *Ehrlichia ruminantium* | pCS20 | MG564186  MG564187  MG564188  MG564189 | AB218277  MG334254 | Sudan, Tanzania, Kenya, Mozambique, Benin | 99.60-100 | 0.003-0.005 |
|  |  | *Theileria lestoquardi* | 18S rRNA | MG564222  MG564223  MG564224  MG564225 | KJ458988  KM117212 | Morocco, Tunisia, China | 99.79-100 | 0.001-0.003 |
|  |  | *Theileria ovis* | 18S rRNA | MG564218  MG564221  MG564219  MG564220 | KF723613  GU726903 | Tunisia, Iran, Turkey | 99.79-100 | 0.002-0.003 |
| Cattle | ***Rhipicephalus annulatus*** | *Anaplasma marginale* | 16S rRNA | MG564242  MG564243  MG564244  MG564245 | AF414872  KU686794 | South Africa, Uganda, Kenya, Italy, Hungary | 99.77-100 | 0.0023-0.0025 |
|  |  | *Babesia bigemina* | 18S rRNA | MG564198  MG564199  MG564200  MG564201 | MH257717  KM076937 | South Africa, Turkey, Bolivia, Brazil | 99.42 | 0.0027-0.0029 |
|  |  | *Babesia bovis* | 18S rRNA | MG564202  MG564203  MG564204  MG564205 | OL305722  KP745628 | Morocco, Turkey, Pakistan | 99.13-100 | 0.003-0.006 |
|  |  | *Borrelia burgdorferi* | 5S-23S ribosomal RNA intergenic spacer region | MG564270  MG564271  MG564272  MG564273 | MK333420  KY594010 | Turkey, China, USA, France | 99.59-100 | 0.003-0.004 |
|  |  | *Borrelia theileri* | *flaB* | MG564190  MG564191  MG564192  MG564193 | MF084761  KX444534 | Republic of Congo, Zambia, Argentina | 98.76-100 | 0.004-0.009 |
|  |  | *Ehrlichia ruminantium* | pCS20 | MG564182  MG564183  MG564184  MG564185 | AB218277  MG334254 | Sudan, Tanzania, Kenya, Mozambique, Benin | 99.60-100 | 0.003-0.005 |
|  |  | *Rickettsia africae* | 16S rRNA | MG564246  MG564247  MG564248  MG564249 | KT633259  KT633255 | Benin, Uganda, Nigeria | 99.75-100 | 0.002-0.006 |
|  |  | *Theileria annulata* | 18S rRNA | MG564206  MG564207  MG564208  MG564209 | MT341858  MN223737 | Egypt, Pakistan, Italy | 100 | Nil |
| Camels | ***Hyalomma dromedarii*** | *Borrelia burgdorferi* | 5S-23S ribosomal RNA intergenic spacer region | MG564266  MG564267  MG564268  MG564269 | MK333420  KY594010 | Turkey, China, USA, France | 99.59-100 | 0.003-0.004 |
|  |  | *Candidatus* Anaplasma camelii | 16S rRNA | MG564234  MG564235  MG564236  MG564237 | MN882725  KX765882 | Saudi Arabia, Iran | 99.71-100 | 0.003-0.007 |
|  |  | *Ehrlichia ruminantium* | pCS20 | MG564178  MG564179  MG564180  MG564181 | AB218277  MG334254 | Sudan, Tanzania, Kenya, Mozambique, Benin | 99.60-100 | 0.003-0.005 |
|  |  | *Mycoplasma arginini* | 16S rRNA | MG564230  MG564231  MG564232  MG564233 | MW493226  KP972459 | Egypt, Turkey, USA | 100 | Nil |
|  |  | *Rickettsia africae* | 16S rRNA | MG564226  MG564227  MG564228  MG564229 | KT633259  KT633255 | Benin, Uganda, Nigeria | 99.75-100 | 0.002-0.006 |
|  |  | *Spiroplasma*-like endosymbiont | 16S rRNA | MG564238  MG564239  MG564240  MG564241 | AB604655  KX559372 | Japan, China | 88.56-100 | 0.005-0.2 |
|  |  | *Theileria annulata* | 18S rRNA | MG564194  MG564195  MG564196  MG564197 | MT341858  MN223737 | Egypt, Pakistan, Italy | 100 | Nil |
|  |  | *Coxiella burnetii* | 16S rRNA | MG564250  MG564253 | MW508465  MW508463 | Tunisia, Indonesia, Japan | 99.72-100 | 0.001-0.002 |
|  |  | *Hepatozoon canis* | 18S rRNA | MG564215  MG564216 | MG077084  MK757802 | Brazil, Turkey, Germany, South Korea | 99.83-100 | 0.001-0.002 |
|  |  | *Leishmania infantum* | ITS1 | MG564278  MG564281 | MK675913  MG969403 | France, Italy, Spain | 100 | Nil |
