# Supplementary material for: Molecular screening and genetic diversity of tick-borne pathogens associated with dogs and livestock ticks in Egypt
Source: PLoS Negl Trop Dis. 2024 Jun 5;18(6):e0012185. doi: 10.1371/journal.pntd.0012185 (PMC11152282; doi:10.1371/journal.pntd.0012185)
Supplement: S3 Table — (DOCX) [file pntd.0012185.s003.docx]

**S3 Table. Symptoms and known impact of detected pathogens of this study.**

| Pathogen/  endosymbiont | Disease | Symptoms/impact | Reports in Egyptian governorates | References |
| --- | --- | --- | --- | --- |
| *Anaplasma marginale* | Bovine anaplasmosis | Destruction of erythrocytes, icterus, anemia, mortality, miscarriage, poor body condition, decreased milk production | Assiut, Beheira, Beni-suef, Cairo, Dakahlia, Damietta, Elminia, Fayoum, Gharbia, Giza, Kafr-Elsheikh, Marsa Matruh, Menofia, New valley, Qena, Qualyobia, Sinai, and Sohag | [1-14] |
| *Candidatus* Anaplasma camelii | NR | NR | Aswan | [15] |
| *Babesia canis vogeli* | Canine babesiosis | anemia, fever, hemoglobinuria, lethargy, and marked thrombocytopenia, death | Cairo, Giza | [11, 37, 48, 67] |
| *Babesia bigemina* | Bovine babesiosis | anemia, fever, hemoglobinuria, lethargy, and marked thrombocytopenia, death | Beheira, Benisuef, Dakahlia, Elminia, Fayoum, Giza, Ismailia, Kafr Elsheikh, Matruh, Menofia, New valley, Qena, Qualyobia, Sharkia, and Sohag | [2, 3, 5-7, 9, 11, 38-43, 45] |
| *Babesia bovis* | Bovine babesiosis | anemia, fever, hemoglobinuria, lethargy, and marked thrombocytopenia, death | Beheira, Benisuef, Dakahlia, Elminia, Fayoum, Giza, Ismailia, Kafr Elsheikh, Matruh, Menofia, New valley, Qena, Qualyobia, Sharkia, and Sohag | [2, 3, 5, 6, 9, 10, 38-42, 44, 46, 47, 68] |
| *Borrelia burgdorferi* | Lyme disease | erythema migrans lesions, chills, fatigue, myalgia, joint pains, headache, fever, swollen lymph nodes, and nervous/heart problems | Fayoum, Beni-Suef, Cairo, and Giza | [16, 26-28] |
| *Borrelia theileri* |  | lethargy, acute anemia, and fever | Cairo, Beni-suef, and Fayoum | [11, 12, 29, 30] |
| *Coxiella burnetii* | Q fever | flu-like, fever, and pneumonia | Alexandria, Aswan, Cairo,  Dakahlia, Giza, Ismailia, Matrouh,  New valley, Port Said, Sharkia,  Sinai | [31-33] |
| *Ehrlichia canis* | Canine ehrlichiosis | fever, lethargy, anorexia, epistaxis, petechial and ecchymosis, depression, lymph adenomegaly, ophthalmic lesions | Alexandria, Cairo, Giza, and Qualyobia | [13, 17-20, 21, 69] |
| *Ehrlichia ruminantium* | Heartwater disease | fever, lethargy, anorexia, epistaxis, petechial and ecchymosis, depression, lymph adenomegaly, and ophthalmic lesions | NR | [70] |
| *Hepatozoon canis* | Canine hepatozoonosis | fever, lethargy, anemia, anorexia, and lymph adenomegaly | Cairo, Giza | [25, 60-63] |
| *Leishmania infantum* | Canine leishmaniasis | anorexia, skin peeling, and dermatitis | NR | [71] |
| *Mycoplasma arginini* | Mycoplasmosis | arthralgia, fever, appetite loss, and pyrexia, respiratory issues | Giza and Menoufiya | [34-36] |
| *Spiroplasma endosymbiont* | Nil | Non-pathogenic | NR | The present study |
| *Rickettsia conorii* | Mediterranean spotted fever (MSF). | fever, rashes, muscular pain, vomiting, diarrhea, and headache | Aswan, Nile Delta,  Nile valley, Sharkia  Cairo, Giza, Sinai | [22,72] |
| *Rickettsia africae* | African tick-bite fever (ATBF) | adenopathy, inoculation eschar, and maculopapular or purpuric rash | Aswan, Nile Delta,  Nile valley, Sharkia  Cairo, Giza, Sinai | [23-25] |
| *Theileria lestoquardi* | Malignant ovine theileriosis | high fever, anorexia, hemolytic anemia, jaundice, hemoglobinuria, lethargy, depression, tachycardia, tachypnea, dyspnea, and diarrhea | Aswan, Beheira, Benisuef, Cairo, Giza, Menofia, New valley, Qualyobia, Sinai, and Upper Egypt | [57-59] |
| *Theileria ovis* | Ovine theileriosis | high fever, anorexia, hemolytic anemia, jaundice, hemoglobinuria, lethargy, depression, tachycardia, tachypnea, dyspnea, and diarrhea | Aswan, Beheira, Benisuef, Cairo, Giza, Menofia, New valley, Qualyobia, Sinai, and Upper Egypt | [57-59] |
| *Theileria annulata* | Tropical theileriosis | high fever, anorexia, hemolytic anemia, jaundice, hemoglobinuria, lethargy, depression, tachycardia, tachypnea, dyspnea, and diarrhea | Aswan, Beheira, Benisuef,  Dakahlia, Elminia, Fayoum, Giza,  Menofia, New valley, Port Said,  Qena, Qualyobia, Sharkia, Sohag  Aswan, Beheira, Benisuef, Cairo,  Giza, Menofia, New valley,  Qualyobia, Sinai | [7, 10, 11, 15, 42, 45, 49, 50-59] |
| *Trypanosoma evansi* | African trypanosomiasis | weakness, abortion in pregnant animals, and weight loss. | Ismaillia, Cairo | [64-66] |

**References:**

1. Younis E, Hegazy NA, El-Deeb W, El-khatib R. Epidemiological and biochemical studies on bovine anaplamosis in Dakahlia and Demiatta governorates in Egypt. Bull Anim Heal Prod Afr. 2010; 57 (4): 12-17. [doi](https://doi): 10.4314/bahpa.v57i4.51668.
2. El-Ashker M, Hotzel H, Gwida M, El-Beskawy M, Silaghi C, Tomaso H. Molecular biological identification of *Babesia*, *Theileria*, and *Anaplasma* species in cattle in Egypt using PCR assays, gene sequence analysis and a novel DNA microarray, Vet Parasitol. 2015; 207: 329–334. [doi: 10.1016/J.VETPAR.2014.12.025](https://doi.org/10.1016/J.VETPAR.2014.12.025).
3. Abou El-Naga T, Barghash S. Blood parasites in camels (*Camelus dromedarius*) in Northern West Coast of Egypt, J Bacteriol Parasitol. 2016; 7: 1–9. [doi: 10.4172/2155-9597.1000258](https://doi.org/10.4172/2155-9597.1000258).
4. Elhariri MT, Elhelw RA, Hamza DA, Soliman DE. Molecular detection of *Anaplasma marginale* in the Egyptian water buffaloes (*Bubalus Bubalis*) based on major surface protein 1α. J Egypt Soc Parasitol. 2017; 47: 247–252.
5. Fereig R, Mohamed S, Mahmoud H, AbouLaila M, Guswanto A, Nguyen T, et al. Seroprevalence of *Babesia bovis*, *B.* *bigemina*, *Trypanosoma evansi*, and *Anaplasma marginale* antibodies in cattle in southern Egypt. Ticks Tick Borne Dis. 2017; 8: 125–131, [doi: 10.1016/J.TTBDIS.2016.10.008](https://doi.org/10.1016/J.TTBDIS.2016.10.008).
6. Al-Hosary A, Răileanu C, Tauchmann O, Fischer S, Nijhof AM, Silaghi C. (2020). Epidemiology and genotyping of *Anaplasma marginale* and co-infection with piroplasms and other Anaplasmataceae in cattle and buffaloes from Egypt. Parasit. Vectors. 131(13): 1–11. <https://doi.org/10.1186/S13071-020-04372-Z>.
7. El-Dakhly, K.M., Arafa, W.M., Soliman, S., Abdel-Fatah, O.R., Wahba, A.A., Esteve-Gasent, M.D., Holman, P.J. Molecular detection, phylogenetic analysis, and genetic diversity of *Theileria annulata*, *Babesia bigemina*, and *Anaplasma marginale* in cattle in three districts of Egypt. Acta Parasitol. 2020; 653(65): 620–627. [doi: 10.2478/S11686-020-00189-Z](https://doi.org/10.2478/S11686-020-00189-Z).
8. Nasreldin N, Ewida R, Hamdon H, Elnaker Y. Molecular diagnosis and biochemical studies of tick-borne diseases (anaplasmosis and babesiosis) in Aberdeen Angus cattle in New Valley. Egypt, Vet World. 2020; 13: 1884–1891. [doi: 10.14202/VETWORLD.2020.1884-1891](https://doi.org/10.14202/VETWORLD.2020.1884-1891).
9. Tumwebaze M, Lee S, Moumouni P, Mohammed-Geba K, Sheir S, Galal K, et al. First detection of *Anaplasma ovis* in sheep and *Anaplasma platys*-like variants from cattle in Menoufia governorate, Egypt. Parasitol Int. 2020; 78: 102150. [doi: 10.1016/J.PARINT.2020.102150](https://doi.org/10.1016/J.PARINT.2020.102150).
10. Al-Hosary A, Răileanu C, Tauchmann O, Fischer S, Nijhof A, Silaghi C. Tick species identification and molecular detection of tick-borne pathogens in blood and ticks collected from cattle in Egypt. Ticks Tick Borne Dis. 2021; 12: 101676. [doi: 10.1016/J.TTBDIS.2021.101676](https://doi.org/10.1016/J.TTBDIS.2021.101676).
11. Abdullah H, Amanzougaghene N, Dahmana H, Louni M, Raoult D, Mediannikov O. Multiple vector-borne pathogens of domestic animals in Egypt. PLoS Negl Trop Dis. 2021a; 15: e0009767. [doi: 10.1371/JOURNAL.PNTD.0009767](https://doi.org/10.1371/JOURNAL.PNTD.0009767).
12. Abdullah H, Aboelsoued D, Farag T, Megeed K, Abdel-Shafy S, Parola P, et al. Molecular characterization of some equine vector-borne pathogens and identification of their vectors in Egypt. Acta Trop. 2021b; 227, 106274, [doi: 10.1016/j.actatropica.2021.106274](https://doi.org/10.1016/j.actatropica.2021.106274).
13. Selim A, Manaa E, Abdelhady A, Ben Said M, Sazmand A. Serological and molecular surveys of *Anaplasma* spp. in Egyptian cattle reveal high *A. marginale* infection prevalence. Iran J Vet Res. 2021; 22: 288. [doi: 10.22099/IJVR.2021.40587.5879](https://doi.org/10.22099/IJVR.2021.40587.5879).
14. Kocan K, Blouin E, Barbet F. Anaplasmosis control: past, present, and future. Ann N Y Acad Sci. 2000; 916: 501–509. [doi: 10.1111/J.1749-6632.2000.TB05329.X](https://doi.org/10.1111/J.1749-6632.2000.TB05329.X).
15. Mohamed WMA, Ali AO, Mahmoud HYAH, Omar MA, Chatanga E, Salim B, et al. Exploring prokaryotic and eukaryotic microbiomes helps in detecting tick-borne infectious agents in the blood of camels. Pathogens. 2021; 10(3): 351. [doi: 10.3390/PATHOGENS10030351](https://doi.org/10.3390/PATHOGENS10030351).
16. Ghafar MW, Eltablawy NA. Molecular survey of five tick-borne pathogens (*Ehrlichia* *chaffeensis*, *Ehrlichia ewingii*, *Anaplasma phagocytophilum*, *Borrelia burgdorferi* sensu lato and *Babesia microti*) in Egyptian farmers. Glob Vet. 2011; 7: 249–255.
17. Botros B, Elmolla M, Salib A, Calamaio C, Dasch G, Arthur R. Canine ehrlichiosis in Egypt: sero-epidemiological survey. Onderstepoort J Vet Res. 1995; 62: 41–43.
18. Salib F, Farghali H. Epidemiological, surgical and therapeutic studies on canine ehrlichiosis in Giza governorate, Egypt. Int J Livestock Res. 2015; 5: 82. [doi: 10.5455/IJLR.20150318092051](https://doi.org/10.5455/IJLR.20150318092051).
19. Salem N, Rakha G, Baraka T. Naturally occurring ehrlichiosis in Egyptian dogs. Iran J Vet Res. 2014; 15: 54–57. [doi: 10.5455/vetworld.2011.522-528](https://doi.org/10.5455/vetworld.2011.522-528).
20. Selim A, Ahmed SS, Galila E. Prevalence and molecular detection of *Ehrlichia canis* in dogs. Benha Vet Med J. 2019; 37: 169–171. [doi: 10.21608/BVMJ.2019.17632.1104](https://doi.org/10.21608/BVMJ.2019.17632.1104).
21. Nasr A, Ghafar M, El Hariri M. Detection of *Anaplasma platys* and *Ehrlichia canis* in *Rhipicephalus sanguineus* ticks attached to dogs from Egypt; a public health concern. Vet Med J. 2020; 66: 1–9. [doi: 10.21608/VMJG.2020.157540](https://doi.org/10.21608/VMJG.2020.157540). (58-78)
22. Raoult D, Zuchelli P, Weiller PJ, Charrel C, San Marco JL, Gallais H, et al. Incidence, clinical observations and risk factors in the severe form of Mediterranean spotted fever among patients admitted to hospital in Marseilles 1983-1984. J Infect. 1986; 12(2): 111-116. [doi: 10.1016/s0163-4453(86)93508-5](https://doi.org/10.1016/s0163-4453(86)93508-5). (85)
23. Raoult D, Fournier PE, Fenollar F, Jensenius M, Prioe T, de Pina JJ, et al. *Rickettsia africae*, a tick-borne pathogen in travelers to sub-Saharan Africa. N Engl J Med. 2001; 344(20): 1504-1510. [doi: 10.1056/NEJM200105173442003](https://doi.org/10.1056/NEJM200105173442003).
24. Bellini C, Monti M, Potin M, Dalle Ave A, Bille J, Greub G. Cardiac involvement in a patient with clinical and serological evidence of African tick-bite fever. BMC Infect Dis. 2005; 5: 90. [doi: 10.1186/1471-2334-5-90](https://doi.org/10.1186/1471-2334-5-90).
25. Johnson EM, Panciera RJ, Allen K., Sheets ME, Beal JD, Ewing SA, et al. Alternate pathway of infection with *Hepatozoon americanum* and the epidemiologic importance of predation. J Vet Intern Med. 2009; 23(6): 1315-8. [doi: 10.1111/j.1939-1676.2009.0375.x](https://doi.org/10.1111/j.1939-1676.2009.0375.x).
26. Adrion ER, Aucott J, Lemke KW, Weiner JP. Health care costs, utilization and patterns of care following Lyme disease. PLoS One. 2015; 10(2): e0116767. [doi: 10.1371/journal.pone.0116767](https://doi.org/10.1371/journal.pone.0116767). (89-92)
27. Elhelw R, El-Enbaawy M, Samir A. Lyme borreliosis: a neglected zoonosis in Egypt. Acta Trop. 2014; 140: 188–192. [doi: 10.1016/J.ACTATROPICA.2014.09.005](https://doi.org/10.1016/J.ACTATROPICA.2014.09.005).
28. Elhelw R, Elhariri M, Hamza D, Abuowarda M, Ismael E, Farag H. Evidence of the presence of *Borrelia burgdorferi* in dogs and associated ticks in Egypt. BMC Vet Res. 2021; 171(17): 1–9. [doi: 10.1186/S12917-020-02733-5](https://doi.org/10.1186/S12917-020-02733-5).
29. Uilenberg G. International collaborative research: significance of tick-borne hemoparasitic diseases to world animal health. Vet Parasitol. 1995; 57: 19–41. [doi: 10.1016/0304-4017(94)03107-8](https://doi.org/10.1016/0304-4017(94)03107-8).
30. Abanda B, Paguem A, Abdoulmoumini M, Kingsley MT, Renz A, Eisenbarth A. Molecular identification and prevalence of tick-borne pathogens in zebu and taurine cattle in North Cameroon. Parasit Vectors. 2019; 12(1): 448. [doi: 10.1186/s13071-019-3699-x](https://doi.org/10.1186/s13071-019-3699-x).
31. Gwida M, El-Ashker M, El-Diasty M, Engelhardt C, Khan I, Neubauer H. Q fever in cattle in some Egyptian Governorates: a preliminary study. BMC Res Notes. 2014; 7: 881. [doi: 10.1186/1756-0500-7-881](https://doi.org/10.1186/1756-0500-7-881).
32. Abushahba MFN, Abdelbaset AE, Rawy MS, Ahmed SO. Cross-sectional study for determining the prevalence of Q fever in small ruminants and humans at El Minya Governorate, Egypt. BMC Res Notes. 2017; 10: 4–9. [doi: 10.1186/s13104-017-2868-2](https://doi.org/10.1186/s13104-017-2868-2).
33. Klemmer J, Njeru J, Emam A, El-Sayed A, Moawad AA, Henning K, et al. Q fever in Egypt: epidemiological survey of *Coxiella burnetii* specific antibodies in cattle, buffaloes, sheep, goats and camels. PLoS One. 2018; 13: e0192188, [doi: 10.1371/JOURNAL.PONE.0192188](https://doi.org/10.1371/JOURNAL.PONE.0192188). (94-100)
34. Hill A. Comparison of mycoplasmas isolated from captive wild felines. Res Vet Sci. 1975; 18(2): 139-45.
35. Goltz JP, Rosendal S, McCraw BM, Ruhnke HL. Experimental studies on the pathogenicity of *Mycoplasma ovipneumoniae* and *Mycoplasma arginini* for the respiratory tract of goats. Can J Vet Res. 1986; 50(1): 59-67.
36. Mousa WS, Zaghawa AA, Elsify AM, Nayel MA, Ibrahim ZH, Al-Kheraije KA, et al. Clinical, histopathological, and molecular characterization of *Mycoplasma* species in sheep and goats in Egypt. Vet World. 2021; 14(9): 2561-2567. [doi: 10.14202/vetworld.2021.2561-2567](https://doi.org/10.14202/vetworld.2021.2561-2567). (109-108)
37. Uilenberg G, Franssen FFJ, Perié NM, Spanjer AAM. Three groups of *Babesia canis* distinguished and a proposal for nomenclature. Vet Q. 1989; 11: 33–40. [doi: 10.1080/01652176.1989.9694194](https://doi.org/10.1080/01652176.1989.9694194). (123)
38. Adham F, Abd-el-Samie E, Gabre R, El-Hussein H. Detection of tick blood parasites in Egypt using PCR assay I–*Babesia bovis* and *Babesia bigemina*, Parasitol Res. 2009; 105: 721–730. [doi: 10.1007/S00436-009-1443-8](https://doi.org/10.1007/S00436-009-1443-8).
39. El-Fayomy AO, Ghoneim AM, Abu-Samak OA, Khidr AA. Contribution of *Babesia* to the illness of cows in Port Said governorate, Egypt. Glob Vet. 2013; 11: 118–122. [doi: 10.5829/idosi.gv.2013.11.1.7453](https://doi.org/10.5829/idosi.gv.2013.11.1.7453).
40. Ibrahim H, Moumouni PA, Mohammed-Geba K, Sheir S, Hashem I, Cao S, et al. Molecular and serological prevalence of *Babesia bigemina* and *Babesia bovis* in cattle and water buffalos under small-scale dairy farming in Beheira and Faiyum provinces, Egypt. Vet Parasitol. 2013; 198: 187–192. [doi: 10.1016/J.VETPAR.2013.08.028](https://doi.org/10.1016/J.VETPAR.2013.08.028). (125-127)
41. Elhaig MM, Selim A, Mahmoud MM, El-Gayar EK. Molecular confirmation of *Trypanosoma evansi* and *Babesia bigemina* in cattle from Lower Egypt. Pak Vet J. 2016; 36: 409–414.
42. Al-Hosary A. Loop-mediated isothermal amplification (LAMP) assay for diagnosis of bovine babesiosis (*Babesia bovis* infection) in Egypt. J Adv Vet Res. 2017; 7: 71–74.
43. Hassan M, Gabr H, Abdel-Shafy S, Hammad K, Mokhtar M. Molecular detection of *Borrelia* spp. in *Ornithodoros savignyi* and *Rhipicephalus annulatus* by *Flab* gene and *Babesia bigemina* in *R. annulatus* by 18S rRNA gene. J Egypt Soc Parasitol. 2017; 47: 403–414. [doi: 10.21608/JESP.2017.77795](https://doi.org/10.21608/JESP.2017.77795).
44. Rizk M, Salama A, El-Sayed S, Elsify A, El-Ashkar M, Ibrahim H, et al. Animal level risk factors associated with *Babesia* and *Theileria* infections in cattle in Egypt, Acta Parasitol. 2017; 62: 796–804. [doi: 10.1515/AP-2017-0096](https://doi.org/10.1515/AP-2017-0096).
45. El-Sayed S, El-Adl M, Ali M, Al-Araby M, Omar M, El-Beskawy M, et al. Molecular detection and identification of *Babesia bovis* and *Trypanosoma* spp. in one-humped camel (*Camelus dromedarius*) breeds in Egypt. Vet World. 2021; 14: 625–633. [doi: 10.14202/VETWORLD.2021.625-633](https://doi.org/10.14202/VETWORLD.2021.625-633).
46. Zaki A, Attia M, Ismael E, Mahdy O. Prevalence, genetic, and biochemical evaluation of immune response of police dogs infected with *Babesia vogeli*, Vet World. 2021; 14: 903–912. [doi: 10.14202/VETWORLD.2021.903-912](https://doi.org/10.14202/VETWORLD.2021.903-912).
47. Nagaty H. Some new and rare records of piroplasmosis with a list of the species of *Babesia* and *Theileria* so far recorded from Egypt. Vet Rec. 1947; 59: 145–147.
48. Hussain S, Hussain A, Ho J, Li J, George D, Sparagano OAE. Tick control in the bovine population of Pakistan: a step towards animal welfare. Universities Federation for Animal Welfare Animal Welfare Conference (UFAW 2021); 2021. (130-137)
49. Ghoneim AM, El-Fayomy AO. Targeting tams-1 gene results in underestimation of *Theileria annulata* infection in diseased cattle in Egypt. Acta Parasitol. 2014; 59: 85–90. [doi: 10.2478/S11686-014-0211-9](https://doi.org/10.2478/S11686-014-0211-9).
50. Al-Hosary AAT, Ahmed L, Seitzer U. First report of molecular identification and characterization of *Theileria* spp. from water buffaloes (*Bubalus bubalis*) in Egypt. Adv Anim Vet Sci. 2015; 3: 629–633. [doi: 10.14737/JOURNAL.AAVS/2015/3.12.629.633](https://doi.org/10.14737/JOURNAL.AAVS/2015/3.12.629.633).
51. Youssef SY, Yasien S, Mousa WMA, Nasr SM, El-Kelesh EAM, Mahran KM, et al. Vector identification and clinical, hematological, biochemical, and parasitological characteristics of camel (*Camelus dromedarius*) theileriosis in Egypt. Trop Anim Health Prod. 2015; 474(47): 649–656. [doi: 10.1007/S11250-015-0771-1](https://doi.org/10.1007/S11250-015-0771-1).
52. Hegab AA, Fahmy MM, Mahdy OA, Wahba A. Parasitological and molecular identification of *Theileria* species by PCR-RFLP method in sheep. Int J Adv Res Biol Sci. 2017; 3: 48–55. [doi: 1.15/ijarbs-2016-3-7-7](http://s-o-i.org/1.15/ijarbs-2016-3-7-7).
53. Al-Hosary A, Ahmed L, Ahmed J, Nijhof A, Clausen P. Epidemiological study on tropical theileriosis (*Theileria annulata* infection) in the Egyptian Oases with special reference to the molecular characterization of *Theileria* spp. Ticks Tick Borne Dis. 2018; 9: 1489–1493. [doi: 10.1016/J.TTBDIS.2018.07.008](https://doi.org/10.1016/J.TTBDIS.2018.07.008).
54. El Damaty HM, Yousef SG, Mahmmod YS, El-Balkemy FA, Mweu MM. Sensitivity and specificity of piroplasm indirect fluorescent antibody test and PCR for *Theileria* *annulata* infection in clinically asymptomatic large ruminants using Bayesian latent class analysis. Vet Parasitol Reg Stud Rep. 2021; 24: 100563. [doi: 10.1016/J.VPRSR.2021.100563](https://doi.org/10.1016/J.VPRSR.2021.100563).
55. Naik BS, Maiti SK, Raghuvanshi PDS. Prevalence of tropical theileriosis in cattle in Chhattisgarh State. J Anim Res. 2016; 6: 1043–1045. [doi: 10.5958/2277-940X.2016.00151.0](https://doi.org/10.5958/2277-940X.2016.00151.0).
56. Khawale TS, Siddiqui MF, Borikar ST, Sakhare MP, Shafi T. Efficacy of arteether against theileriosis in cattle. J Anim Res. 2019; 9(6): 883-888.
57. Gebrekidan H, Perera PK, Ghafar A, Abbas T, Gasser RB, Jabbar A. An appraisal of oriental theileriosis and the *Theileria orientalis* complex, with an emphasis on diagnosis and genetic characterisation. Parasitol Res. 2020; 119(1): 11-22. [doi: 10.1007/s00436-019-06557-7](https://doi.org/10.1007/s00436-019-06557-7).
58. Vincent-Johnson N. (2014). Canine and feline hepatozoonosis. In: Saunders WB, editor. Canine and feline infectious diseases. New York: Academic Press; 2014. pp. 747–759. [doi: 10.1016/B978-1-4377-0795-3.00077-6](https://doi.org/10.1016/B978-1-4377-0795-3.00077-6).
59. Baneth G, Samish M, Shkap V. Life cycle of *Hepatozoon canis* (Apicomplexa: Adeleorina: Hepatozoidae) in the tick *Rhipicephalus sanguineus* and domestic dog (*Canis familiaris*). J Parasitol. 2007; 93(2): 283-99. [doi: 10.1645/GE-494R.1](https://doi.org/10.1645/GE-494R.1). (140-150)
60. El Hindawy MR. Studies on the blood of dogs: VI. Haematological findings in some diseases caused by specific blood parasites: (a) *Babesia canis*; (b) *Hepatozoon canis*. Br Vet J. 1951; 107: 303–309. [doi: 10.1016/S0007-1935(17)52110-7](https://doi.org/10.1016/S0007-1935(17)52110-7).
61. Hegab AA, Fahmy MM, Omar HM, Abuowarda MM, Gattas SG. Investigation of tickborne pathogens within naturally infected brown dog tick (Ixodidae: *Rhipicephalus* *sanguineus*) in Egypt by light and electron microscopy. Int J Vet Sci. 2020; 9: 476–482. [doi: 10.37422/IJVS/20.064](https://doi.org/10.37422/IJVS/20.064).
62. Dantas–Torres F. Ticks as vectors of *Leishmania* parasites. Trend Parasitol. 2011; 77(4): 155–160. [doi: 10.1016/j.pt.2010.12.006](http://doi.org/10.1016/j.pt.2010.12.006).
63. Hilali M, Abdel-Gawad A, Nassar A, Abdel-Wahab A, Magnus E, Büscher P. Evaluation of the card agglutination test (CATT/*T. evansi*) for detection of *Trypanosoma* *evansi* infection in water buffaloes (*Bubalus bubalis*) in Egypt. Vet Parasitol. 2004; 121(1-2): 45-51. [doi: 10.1016/j.vetpar.2004.02.009](https://doi.org/10.1016/j.vetpar.2004.02.009). (152-155)
64. Pourjafar M, Badiei K, Sharifiyazdi H, Chalmeh A, Naghib M, Babazadeh M, et al. Genetic characterization and phylogenetic analysis of *Trypanosoma evansi* in Iranian dromedary camels. Parasitol Res. 2013; 112(2): 899-903. [doi: 10.1007/s00436-012-3121-5](https://doi.org/10.1007/s00436-012-3121-5).
65. Ventura RM, Takeda GF, Silva RA, Nunes VL, Buck GA, Teixeira MM. Genetic relatedness among *Trypanosoma evansi* stocks by random amplification of polymorphic DNA and evaluation of a synapomorphic DNA fragment for species-specific diagnosis. Int J Parasitol. 2002; 32(1): 53-63. [doi: 10.1016/s0020-7519(01)00314-9](https://doi.org/10.1016/s0020-7519(01)00314-9).
66. Nakayima J, Nakao R, Alhassan A, Mahama C, Afakye K, Sugimoto C. Molecular epidemiological studies on animal trypanosomiases in Ghana. Parasit Vectors. 2012; 5: 217. [doi: 10.1186/1756-3305-5-217](https://doi.org/10.1186/1756-3305-5-217). (157-159)
67. Salem NY, Farag HS. Clinical, hematologic, and molecular findings in naturally occurring *Babesia canis vogeli* in Egyptian dogs, Vet Med Int. 2014; 270345. [doi: 10.1155/2014/270345](https://doi.org/10.1155/2014/270345).
68. Mahmmod Y. Natural *Babesia bovis* infection in water buffaloes (*Bubalus bubalis*) and crossbred cattle under field conditions in Egypt: a preliminary study. J Arthropod Borne Dis. 2013; 8: 1–9.
69. Komnenou AA, Mylonakis ME, Kouti V, Tendoma L, Leontides L, Skountzou E, et al. Ocular manifestations of natural canine monocytic ehrlichiosis (*Ehrlichia canis*): a retrospective study of 90 cases. Vet Ophthalmol. 2007; 10(3): 137-42. [doi: 10.1111/j.1463-5224.2007.00508.x](https://doi.org/10.1111/j.1463-5224.2007.00508.x).
70. Van de Pypekamp HE, Prozesky L. Heartwater. An overview of the clinical signs, susceptibility and differential diagnoses of the disease in domestic ruminants. Onderstepoort J Vet Res. 1987; 54(3):263-266.
71. Koutinas AF, Koutinas CK. Pathologic mechanisms underlying the clinical findings in canine leishmaniasis due to *Leishmania infantum*/*chagasi*. Vet Pathol. 2014; 51(2): 527-538. [doi: 10.1177/0300985814521248](https://doi.org/10.1177/0300985814521248).
72. Latifian M, Khalili M, Farrokhnia M, Mostafavi E, Esmaeili S. *Rickettsia conorii* subsp. *israelensis* infection: a case report from southeast Iran. BMC Infect Dis. 2022; 22(1): 320. [doi](https://doi): 10.1186/s12879-022-07291-9. (182-187)
