# Supplementary figures and images for: Molecular screening and genetic diversity of tick-borne pathogens associated with dogs and livestock ticks in Egypt
Source: PLoS Negl Trop Dis. 2024 Jun 5;18(6):e0012185. doi: 10.1371/journal.pntd.0012185 (PMC11152282; doi:10.1371/journal.pntd.0012185)

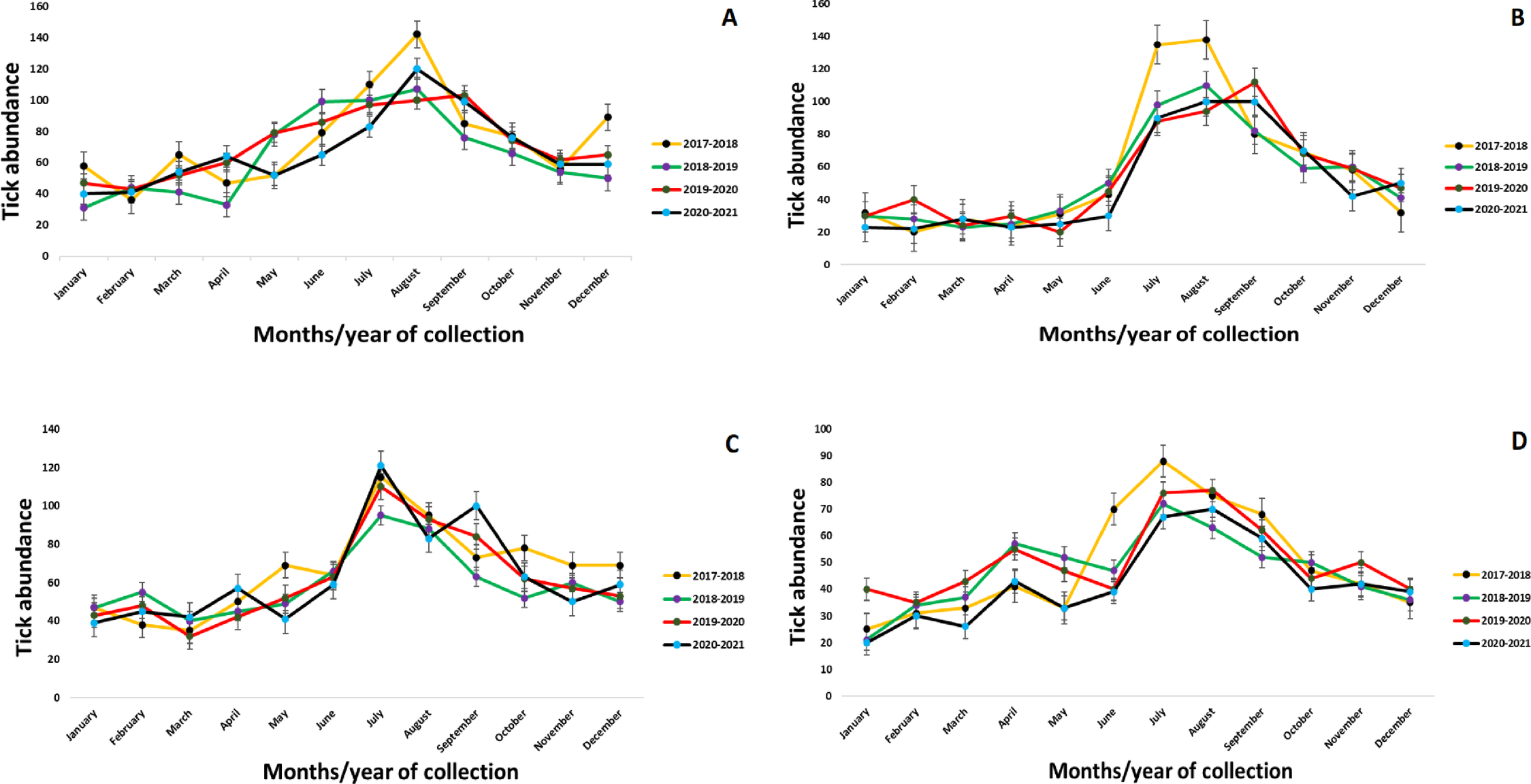

Supplement: S1 Fig — Species-wise amplitude of ticks during the collection trips; (A) Rhipicephalus rutilus (Dogs), (B) Rh. rutilus (Sheep), (C) Rh. annulatus, (D) Hyalomma dromedarii. (TIF) [file pntd.0012185.s004.tif]

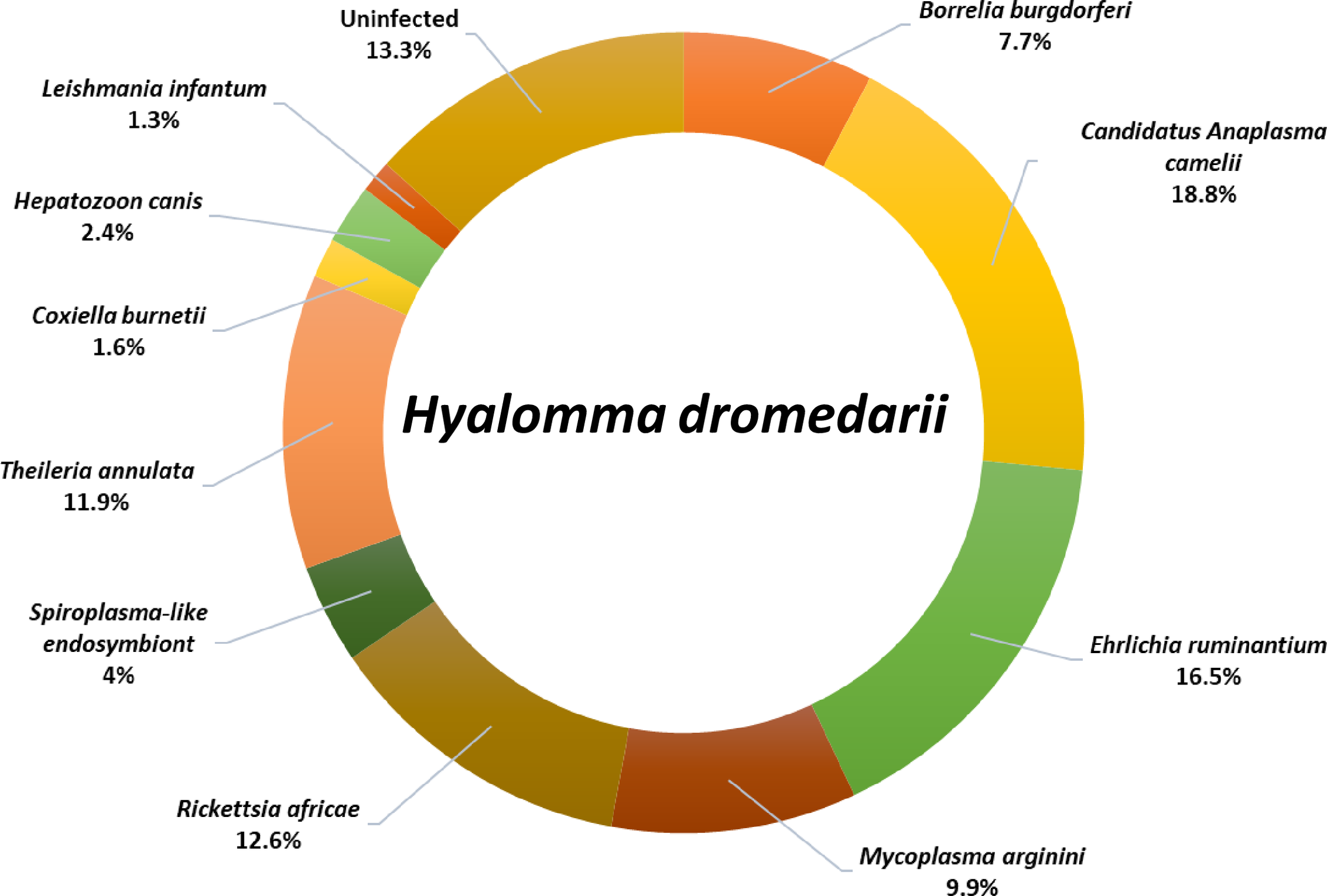

Supplement: S2 Fig — (TIF) [file pntd.0012185.s005.tif]

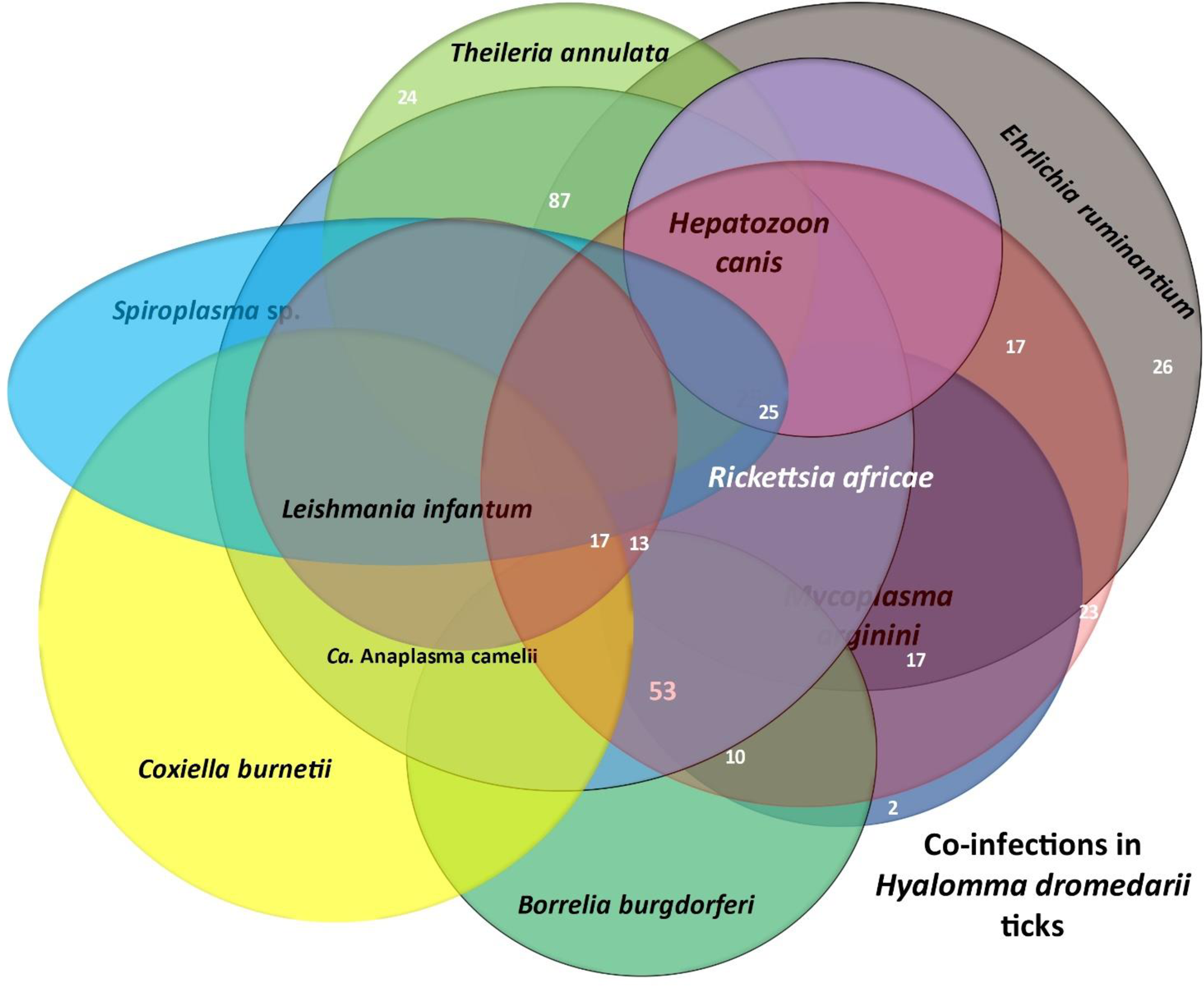

Supplement: S3 Fig — (TIF) [file pntd.0012185.s006.tif]

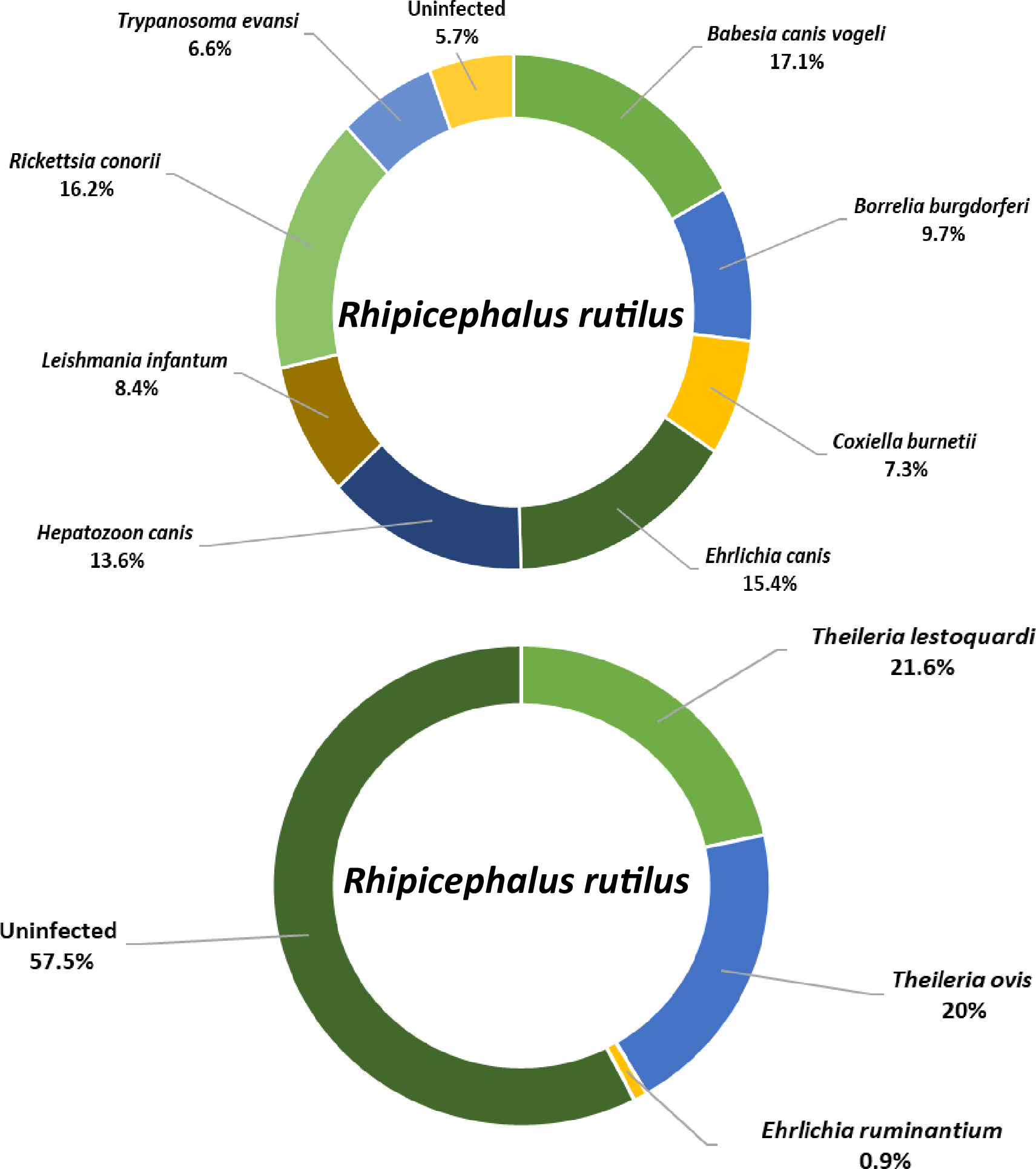

Supplement: S4 Fig — Pathogens’ prevalence detected in Rhipicephalus rutilus ticks infesting dogs (top) and sheep (bottom) of the present study (The percentages are out of 865 and 570 screened ticks of dogs and sheep, respectively). (TIF) [file pntd.0012185.s007.tif]

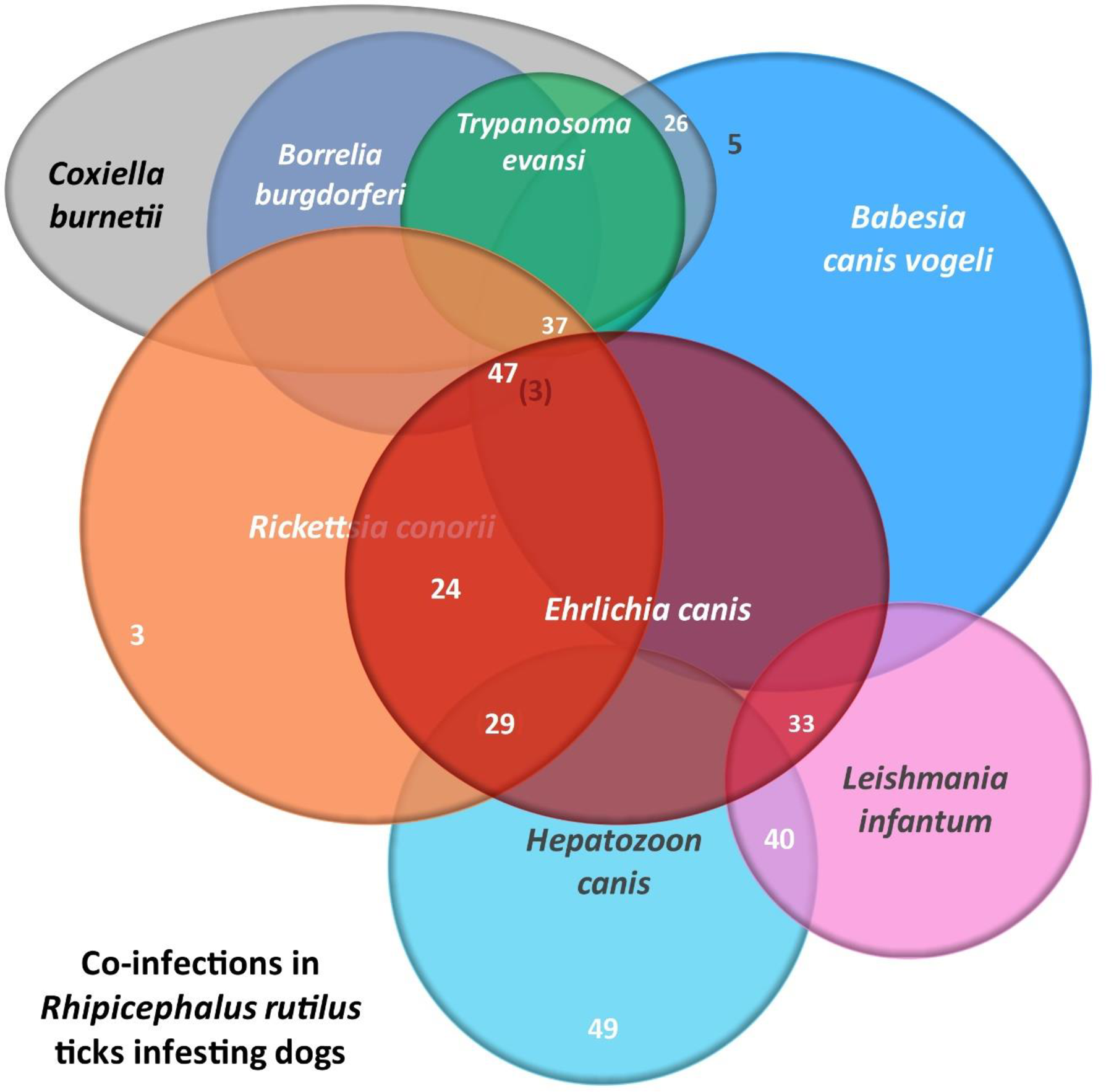

Supplement: S5 Fig — (TIF) [file pntd.0012185.s008.tif]

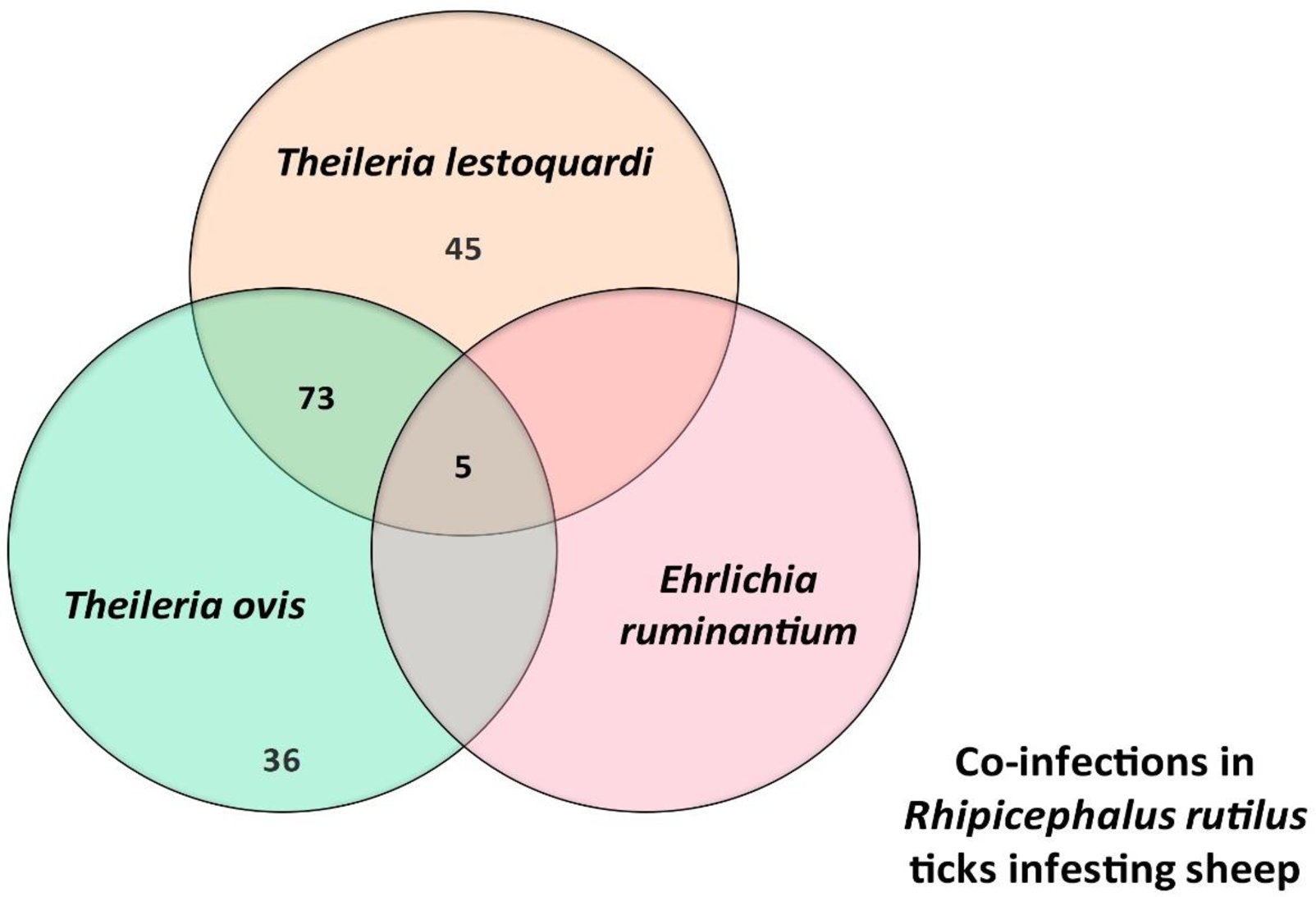

Supplement: S6 Fig — (TIF) [file pntd.0012185.s009.tif]

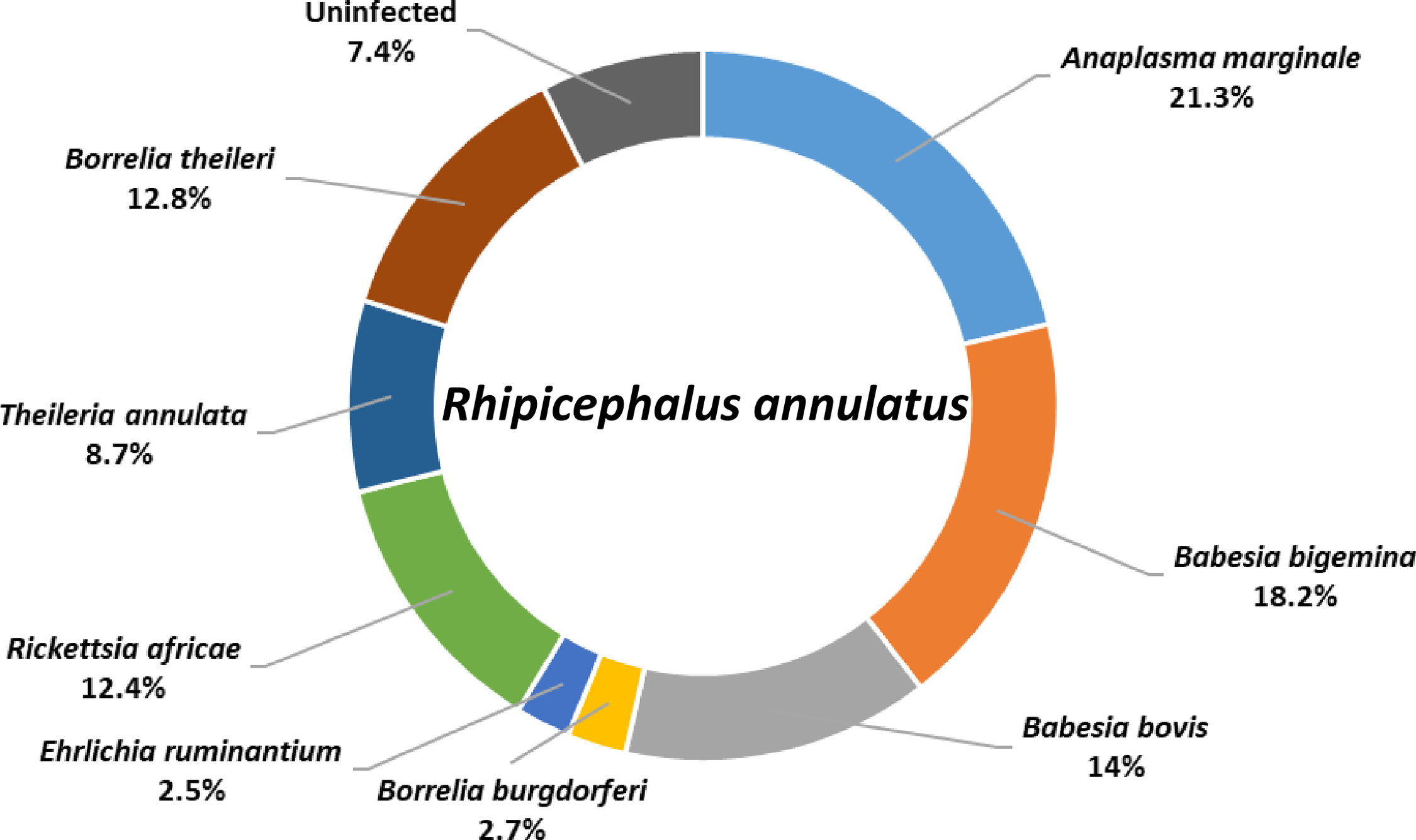

Supplement: S7 Fig — (TIF) [file pntd.0012185.s010.tif]

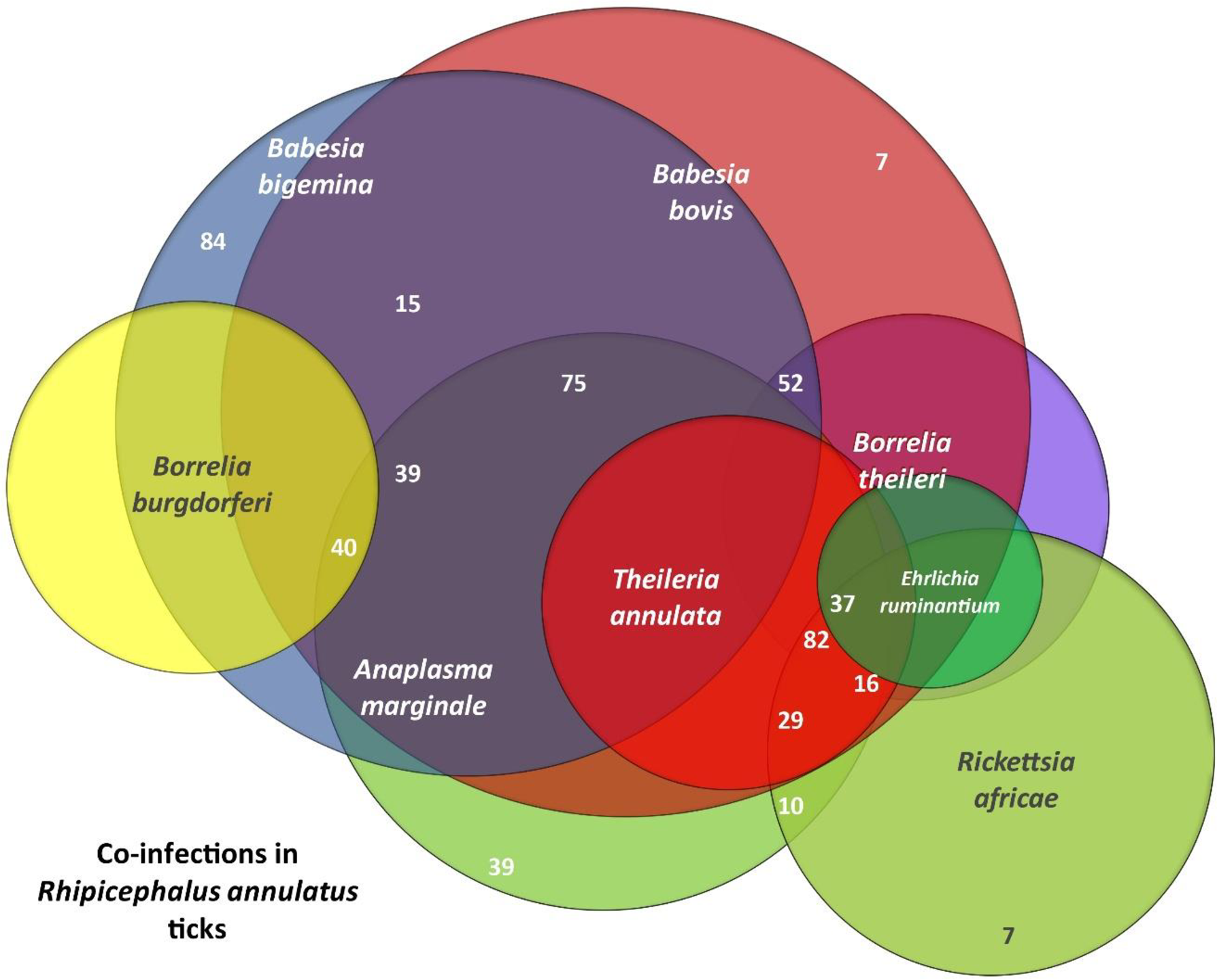

Supplement: S8 Fig — (TIF) [file pntd.0012185.s011.tif]
